# Therapeutic evaluation of palbociclib and its compatibility with other chemotherapies for primary and recurrent nasopharyngeal carcinoma

**DOI:** 10.1186/s13046-020-01763-z

**Published:** 2020-11-26

**Authors:** Zhichao Xue, Vivian Wai Yan Lui, Yongshu Li, Lin Jia, Chanping You, Xin Li, Wenying Piao, Hui Yuan, Pek Lan Khong, Kwok Wai Lo, Lydia Wai Ting Cheung, Victor Ho Fan Lee, Anne Wing Mui Lee, Sai Wah Tsao, Chi Man Tsang

**Affiliations:** 1 School of Biomedical Sciences, Li Ka Shing Faculty of Medicine, The University of Hong Kong, Hong Kong Special Administrative Region, China; 2 School of Biomedical Sciences, Faculty of Medicine, The Chinese University of Hong Kong, Hong Kong Special Administrative Region, China; 3 Department of Anatomical and Cellular Pathology and State Key Laboratory of Translational Oncology, The Chinese University of Hong Kong, Hong Kong Special Administrative Region, China; 4 Shenzhen Key Laboratory of Viral Oncology, The Clinical Innovation & Research Center (CIRC), Shenzhen Hospital, Southern Medical University, Shenzhen, China; 5 Department of Diagnostic Radiology, Faculty of Medicine, The University of Hong Kong, Hong Kong Special Administrative Region, China; 6 Guangdong Academy of Medical Sciences and Guangdong Provincial People’s Hospital, Guangzhou, Guangdong PR China; 7 Department of Clinical Oncology, Li Ka Shing Faculty of Medicine, The University of Hong Kong, Hong Kong Special Administrative Region, China

**Keywords:** Nasopharyngeal carcinoma, Patient-derived xenografts, Palbociclib, SAHA, Drug resistance

## Abstract

**Background:**

Recent genomic analyses revealed that druggable molecule targets were only detectable in approximately 6% of patients with nasopharyngeal carcinoma (NPC). However, a dependency on dysregulated CDK4/6–cyclinD1 pathway signaling is an essential event in the pathogenesis of NPC. In this study, we aimed to evaluate the therapeutic efficacy of a specific CDK4/6 inhibitor, palbociclib, and its compatibility with other chemotherapeutic drugs for the treatment of NPC by using newly established xenograft models and cell lines derived from primary, recurrent, and metastatic NPC.

**Methods:**

We evaluated the efficacies of palbociclib monotherapy and concurrent treatment with palbociclib and cisplatin or suberanilohydroxamic acid (SAHA) in NPC cell lines and xenograft models. RNA sequencing was then used to profile the drug response–related pathways. Palbociclib-resistant NPC cell lines were established to determine the potential use of cisplatin as a second-line treatment after the development of palbociclib resistance. We further examined the efficacy of palbociclib treatment against cisplatin-resistant NPC cells.

**Results:**

In NPC cells, palbociclib monotherapy was confirmed to induce cell cycle arrest in the G1 phase in vitro. Palbociclib monotherapy also had significant inhibitory effects in all six tested NPC tumor models in vivo, as indicated by substantial reductions in the total tumor volumes and in Ki-67 proliferation marker expression. In NPC cells, concurrent palbociclib treatment mitigated the cytotoxic effect of cisplatin in vitro. Notably, concurrent treatment with palbociclib and SAHA synergistically promoted NPC cell death both in vitro and in vivo. This combination also further inhibited tumor growth by inducing autophagy-associated cell death. NPC cell lines with induced palbociclib or cisplatin resistance remained sensitive to treatment with cisplatin or palbociclib, respectively.

**Conclusions:**

Our study findings provide essential support for the use of palbociclib as an alternative therapy for NPC and increase awareness of the effective timing of palbociclib administration with other chemotherapeutic drugs. Our results provide a foundation for the design of first-in-human clinical trials of palbociclib regimens in patients with NPC.

**Supplementary Information:**

**Supplementary information** accompanies this paper at 10.1186/s13046-020-01763-z.

## Background

Nasopharyngeal carcinoma (NPC) is a prevalent malignancy in southern China and throughout Southeast Asia [1]. Currently, a combination of platinum-based chemotherapy and radiotherapy is the mainstay of treatment for primary and local NPC [1]. However, 5–10% of all patients and 15–45% of those with stage IV NPC develop locally recurrent disease after treatment, and approximately a quarter of patients in the latter group also develop distant metastases [2, 3]. Accordingly, the management of advanced NPC, including recurrent, metastatic, and chemoresistant tumors, remains a major challenge. New targeted therapeutic methods that can effectively control NPC at both early and advanced stages of disease are highly sought after as a means of improving survival outcomes.

Currently, the screening and evaluation of novel and potentially effective therapeutic agents against NPC is significantly limited by the lack of a preclinical NPC model. NPC patient-derived xenografts and cell lines are difficult to establish. Although a few such xenografts have been developed for investigation, including XenoC15 and XenoC17 (derived from African NPC patients [4];) and Xeno2117 and Xeno666 (derived from a Hong Kong NPC patient [5];), all were established more than 25 years ago and have been passaged continuously in nude mice. As a result, the genetic and pathological properties of these xenografts may have diverged from those of the parental NPC tumors isolated from patients. Only one in vitro Epstein–Barr virus (EBV)–positive NPC cell line (C666–1) is available for investigation [6], whereas other commonly used “NPC cells” have lost their EBV episomes and may not be representative of NPC [7]. Furthermore, the detection of HeLa cell’s and HPV18’s genomic materials in these cell lines has cast doubt on the cellular origins [7] and has limited the applications of these lines in evaluations of novel therapeutic agents against NPC. To address this limitation, we have established new NPC xenografts and cell lines for in vivo and in vitro investigations, including Xeno32 and Xeno76 (xenografts derived from primary NPC [8];), Xeno23 and NPC43 (a xenograft and cell line respectively derived from recurrent NPC [8];) and C17 (NPC cell line derived from a xenograft of metastatic NPC [9];). Together with the conventional NPC cell line C666–1, these newly established NPC xenografts and cell lines represent a comprehensive panel of preclinical NPC models available to assess chemotherapeutic drug efficacy.

The field of targeted therapy for NPC is grossly underdeveloped due to the scarcity of representative preclinical NPC models for novel agent evaluations and our insufficient knowledge of the genomic properties of NPC. Our group and others recently conducted several genomic analyses to define the genetic alterations that contribute to NPC tumorigenesis [10–12] and generated knowledge that will shape the focus of future strategies for NPC therapeutic development [13]. Currently, the genetic alterations amenable to targeted therapies are heterogenous and present at relatively low rates in NPC. Specifically, mutations in *PIK3CA*, *EGFR*, *FGFR1/2/3/4*, and *BRCA1*/*BRCA2*/*ATM* were identified in only 1.68, 0.24, 2.16, and 1.68% of patients with NPC, respectively [10, 11, 14, 15].

Research evidence suggests that the aggressive growth and metastatic behaviors of cancer cells depend on the dysregulation of p16–CDK4/6–cyclin D1–RB signaling. In proliferating cells, the suppression of p16 expression relieves the inhibitory effect of this protein on the kinase activity of CDK4/6. The CDK4/6 kinases then form an active complex with cyclin D, which hyper-phosphorylates RB and releases E2F to initiate a cascade of downstream events involved in the transcription of proliferation genes. This process enables the cell to enter the cell cycle. The overexpression of cyclin D1 and downregulation of p16 are common events in NPC, and therefore treatment with a specific CDK4/6 inhibitor could target this essential cell cycle regulatory pathway and improve the druggability of NPC [16–18]. Previously, we detected cyclin D1 overexpression in more than 90% of NPC tumor tissues [18]. Cyclin D1 is also overexpressed in premalignant and dysplastic nasopharyngeal epithelial (NPE) cells and may play an important role in early NPC pathogenesis by supporting persistent and latent EBV infection in the premalignant nasopharyngeal epithelium [19]. A whole-exome sequencing analysis of a large cohort of patients with NPC (*N* > 100) also identified cyclin D1 amplification and homozygous p16 gene deletion as common features of NPC [10]. Importantly, the observation that RB mutation is uncommon in NPC suggests that a blockade of CDK4/6 activity could abolish the dependency of this tumor type on the cyclin D1–RB pathway.

In this study, we used a comprehensive panel of NPC models to examine the efficacy of palbociclib, a selective CDK4/6 inhibitor approved by the Food and Drug Administration (FDA), as a treatment for NPC. Palbociclib was initially tested in both in vitro and in vivo models of breast cancer and was shown to effectively inhibit the growth of tumor cells, especially cell lines with increased RB phosphorylation and cyclin D1 expression and decreased p16 expression [20]. Later, patients with breast cancer were recruited to participate in the human clinical trial PALOMA-2, which demonstrated that palbociclib treatment could prolong the median progression-free survival duration for more than 10 months. In 2016, palbociclib was approved by the FDA for the treatment of hormone receptor–positive, human epidermal growth factor receptor 2–negative advanced or metastatic breast cancer [21]. The results of preclinical studies in other cancer types, such as hepatocellular carcinoma [22], ovarian cancer [23], rhabdoid tumor [24], and glioblastoma [25], also indicated that palbociclib effectively targets cancer cells with the following characteristics: (a) cyclin D1 overexpression, (b) functional RB, and (c) p16 inactivation. As described above, the CDK4/6–cyclinD1 axis is often dysregulated in NPCs at all stages, so inhibition of the related activity represents a common target in primary, recurrent, and even metastatic NPC.

In this study, we also examined the therapeutic efficacy of combinations of palbociclib with two other FDA-approved chemotherapeutic drugs, cisplatin and suberanilohydroxamic acid (SAHA). Antagonistic effect was observed in vitro when palbociclib and cisplatin were used together to treat the NPC cells. Interestingly, we observed a synergistic effect when SAHA was combined with palbociclib. SAHA is a histone deacetylase (HDAC) inhibitor that alters gene transcription by inhibiting histone deacetylation and inducing chromatin relaxation, leading to the general expression of genes that encode tumor suppressors [26]. The synergistic effects of combined treatment with SAHA and palbociclib were also confirmed for the first time in our preclinical NPC xenograft models. Furthermore, transcriptome profiling of SAHA- and palbociclib-treated NPC cells revealed that the synergistic inhibitory actions of these drugs on NPC cells may be related to the activation of autophagy. Finally, we established palbociclib- and cisplatin-resistant cell lines and evaluated the responses of these cells to cisplatin and palbociclib, respectively. Our findings provide essential information to support the design of the first in-human trial of palbociclib therapy for the treatment of NPC.

## Materials and methods

### Non-malignant NPE and cancerous NPC cell lines

Three telomerase-immortalized, nonmalignant human NPE cell lines (NP361 and NP460) [19, 27] and one SV40T-immortalized NPE cell line (NP69) [28] were used as non–cancer cell controls in this study. All NPE cell lines were cultured under the conditions described in our previous publication [29]. Three EBV-positive NPC cell lines (C666–1, NPC43, and C17) were also used in this study. C666–1 [6] was established from an NPC xenograft, XenoC666, which is widely used in preclinical studies on NPC. NPC43 [8] and C17 [9] were newly established in our laboratory and have been carefully characterized with respect to EBV infection status, genomic profiles, and growth properties. The NPC cell lines were maintained as monolayers in RPMI supplemented with 10% FBS and 1% penicillin/streptomycin, and their responses to palbociclib were evaluated under both two-dimensional (2-D; monolayer) and 3-D (spheroid) culture conditions. For 3-D culture, the NPC cells were seeded in ultra-low attachment plates (Corning, #4520) to enable the formation of floating spheroids.

### NPC xenografts

Four-week-old male immunodeficient (NOD/SCID) mice were supplied by the Laboratory Animal Unit (LAU) of The Hong Kong University (HKU) and housed under pathogen-free conditions. All animal experiments were conducted according to the animal license issued by the Hong Kong Department of Health and with the approval of the Committee on the Use of Live Animals in Teaching and Research (CULATR) of HKU. To initiate the growth of NPC cell lines (C17, NPC43, and C666–1 cells) as tumor xenografts in NOD/SCID mice, we resuspended 10^7^ cells in 200 μl of a 1:1 (vol/vol) mixture of Matrigel and culture medium and injected this suspension subcutaneously into the left dorsal flank region of each mouse.

For the three newly established xenografts (Xeno23, Xeno32, and Xeno76) [8], xenografted tumors were cut into 2 mm^3^ blocks and implanted subcutaneously into the left dorsal flank regions of mice. The mice were then randomized into drug treatment or vehicle control groups once the tumors became palpable (i.e., 4-mm diameter). The CDK4/6 inhibitor palbociclib (Pfizer, 571,190–30-2) was dissolved in filtered distilled deionized water (ddH_2_O; 7.5 mg/ml) and administered daily to mice via oral gavage at the concentrations stated for each experiment. SAHA (Cayman Chemical, 10,009,929) was dissolved in DMSO to a concentration of 100 mg/ml and administered by intraperitoneal injection (20 μl per mouse). The tumor size and animal body weight were recorded every other day throughout the treatment period. All mice were euthanized at the end of the experiment, and the tumors were excised and fixed with 10% neutral buffered formalin (NBF) before histopathological and immunohistochemical examinations.

For the metastasis model, each NOD/SCID mouse was injected with 10^6^ C666–1 cells via the tail vein, and treatment was initiated 10 days later. The mice were dosed with vehicle or palbociclib continuously for 21 days and left for observation for 124 days. At the end of the study, lung tissues were dissected from the mice, fixed, and processed to examine the presence of growth of C666–1 cells.

### Cell viability determination

NPC cells in culture medium were seeded at a density of 4000 cells/well in a 96-well plate (100 μl per well) and incubated overnight. Palbociclib (Selleck Chemicals, S1116) was dissolved in cell culture medium to concentrations of 0–20 μM and added to the NPC cell cultures. The viability of the cells was examined on Days 1, 3 and 5 of culture. SAHA was diluted from a stock solution (100 mg/ml) into culture medium and used at various treatment concentrations (0–0.5 μM). The concentration of DMSO (vehicle) was less than 0.001% in the culture with the highest concentration of SAHA. Cisplatin (Sigma, 479,306) was diluted with dimethylformamide (DMF) to a stock concentration of 40 mM.

A resazurin (Sigma, R7017) stock solution (0.02% w/v dissolved in PBS) was added to the cells (10% v/v) to determine cell viability after drug treatment. After incubation with resazurin for 4 h, the amount of the fluorescent product resorufin in the cultures was measured at excitation/emission wavelengths of 530/590 nm using a Victor 3 Plate Reader (PerkinElmer). Growth inhibition in each well was calculated as: (viability_control_-viability_drug_) / viability_control_ *100%.

### Western blot

RIPA lysis buffer [50 mM Tris-HCl (pH 8.0), 150 mM NaCl, 1% Nonidet P40, 0.5% deoxycholic acid, 0.1% sodium dodecyl sulfate (SDS)] was supplemented with protease inhibitors (1 tablet/10 ml RIPA buffer; Thermo Scientific, A32961) and PhosSTOP (1 tablet/10 ml RIPA buffer; Roche, 4,906,837,001) immediately prior to use. After lysing the cells in RIPA buffer, the protein concentrations in the samples were measured and adjusted to ensure that equal amounts of protein per sample would be resolved by SDS–polyacrylamide gel electrophoresis. The resolved proteins were then transferred from the gels to PVDF membranes (GE Healthcare, 10,500,023).

The following specific antibodies were used to detect various proteins in the membrane-bound samples: RB (1:1000 dilution; BD, 554136), phosphorylated (phospho)-RB (Ser708) (1:1000; Cell Signaling Technology, 9307S), cyclin A (1:1000; Santa Cruz, sc-751), cyclin B1 (1:1000; Cell Signaling Technology, 4138), cyclin D1 (1:1000; BD Biosciences, 556,470), cyclin D2 (1:1000; BD Biosciences, 3741), cyclin D3 (1:1000; BD Biosciences, 2936), cyclin E1 (1:1000; Cell Signaling Technology, 4129), cyclin E2 (1:1000; Cell Signaling Technology, 4132), Cyclin H (1:1000; BD Biosciences, 2927), cleaved PARP (1:1000; Cell Signaling Technology, 9541S), CDK2 (1:1000; Santa Cruz, SC6248), CDK4 (1:1000; Santa Cruz, SC260), CDK6 (1:1000; Santa Cruz, SC271364), E-cadherin (1:1000; Santa Cruz, SC21791), N-cadherin (1:1000; Santa Cruz, SC7939), caspase 3 (1:1000; Cell Signaling Technology,14,220), cleaved caspase-3 (1:1000; Cell Signaling Technology, 9664), PARP (1:1000; Santa Cruz, SC8007), cleaved PARP (1:1000; Cell Signaling Technology, 9541S), involucrin (1:1000; Thermo Fisher, MS-126-P1ABX), Bcl-2 (1:1000; Cell Signaling Technology, 15071S), Bax (1:1000; Cell Signaling Technology, 5023S), and LC3 (1:1000; Cell Signaling Technology, 2775S). The chemiluminescence signal corresponding to each labeled protein was then captured with the ChemiDoc MP Imaging System (Bio-Rad). A specific antibody against GAPDH (1:10000; Proteintech, 10,494–1-AP) was used as a protein loading control.

### Cell cycle analysis

Cells were detached from the plates using trypsin, washed with cold PBS, and fixed in 70% ethanol at 4 °C overnight. The cells were then washed with PBS and incubated with propidium iodide (1 μg/ml; Invitrogen, P3566) and RNase (10 μg/ml; Roche, 10,109,169,001) for 30 min. After washing, 10^4^ cells per sample were analyzed on a FACSCalibur flow cytometer (BD Biosciences) to detect the DNA content. FlowJo software (TreeStar) was used for the data analysis.

### Histology and immunohistochemistry

Fresh tumor samples collected from NOD/SCID mice were fixed in 10% neutral buffered formalin, embedded in paraffin and processed for immunohistochemical examinations. Five-micrometer-thick sections were cut from embedded tumors and baked in a 37 °C oven overnight before processing for hematoxylin–eosin and immunohistochemical staining.

For immunohistochemical staining, the tissues were subjected to antigen retrieval by boiling in sodium citrate buffer (10 mM, pH 6.0), followed by incubation at a temperature just below boiling for 20 min. The paraffin sections on slides were then de-waxed and rehydrated. The sections were incubated with 3% H_2_O_2_ for 8 min to inactivate endogenous peroxidases and then with 3% bovine serum albumin for another 8 min to block non-specific protein binding sites. The following specific antibodies were used for immunohistochemical analyses: AE1/AE3 for keratin detection (1:250; DAKO, M3515) and Ki-67 as a cell proliferation marker (1:200; Santa Cruz, sc-23,900). After overnight incubation with the primary antibody in a moist chamber, a horseradish peroxidase-conjugated secondary antibody (DAKO, K4001) was applied to the sections for 1 h at room temperature. Finally, 3,3′-diamino-benzidine substrate (DAB; DAKO, K346711–2) was applied to the sections for brown color development. The slides were then dehydrated, mounted with Permount mounting medium (DAKO, S3023), and scanned and analyzed using a Vectra Polaris Imaging System (Perkin Elmer).

### RNAscope detection of EBV gene expression in NPC xenografts

An RNA in situ hybridization protocol was conducted to examine the expression of the selected EBV gene BZLF1 (Advanced Cell Diagnostic, 450,411). For this analysis, the RNAscope 2.5HD detection kit (Advanced Cell Diagnostic, 322,370) was used according to the manufacturer’s recommended protocol. This new RNA-in situ hybridization platform uses a specific and sensitive RNA-FISH probe provided by the company to detect specific EBV-RNA expression during lytic reactivation.

### RNA sequencing analysis

To evaluate the RNA profiles of NPC cells after drug treatments, mRNA libraries were prepared using the TruSeq mRNA Library Prep kit (Illumina) and sequenced on a HiSeq2000 system (Illumina). The gene expression ratio between the treatment groups was then calculated based on the fragments per kilobase of transcript per million mapped reads of each gene (FPKM). HISAT2 was used to perform a sequence alignment analysis based on the reference sequence [30], and the alignment results were inputted into Stringtie [31] to complete the quantitative gene expression analysis. The differential expression analysis was conducted using edgeR [32]. Kyoto Encyclopedia of Genes and Genomes (KEGG) and Gene Ontology (GO) enrichment analyses were conducted using ClusterProfiler.

### Micro-positron emission tomography (PET)/magnetic resonance imaging (MRI) scanning

The mice were scanned using a nanoScan PET/3 T MRI scanner (Mediso) with spatial resolutions of 700 μm for PET and 100 μm for MRI. Before scanning, the mice were fasted overnight. Sixty minutes before the PET/MRI scan, each mouse received an injection of 200 μCi of ^18^F-flurodeoxyglucose (FDG) via the tail vein with < 2 min of isoflurane inhalation (5% in 100% oxygen). For the scan, each mouse was placed into the PET/MRI scanner in a head holder and remained under isoflurane inhalation (2% in 100% oxygen) until the end of the scan. The body temperature and respiration rate were monitored throughout the scan. The FDG uptake was quantified using standardized uptake values (SUVs), which were calculated using the following formula: ¼ regional FDG concentration (Bq/mL)/injected FDG dose (Bq)* body weight (kg). The raw images were anatomically standardized to achieve a symmetrical midline alignment. The images were then reconstructed using Nucline software (Mediso), and PET/MRI fused images were coregistered using InterView FUSION (Mediso). The internal liver metabolism (SUV_liver_ = 0.5) was used as the basal metabolism level. The SUV of the tumor (SUV_tumor_) was normalized to the basal metabolism. SUV=SUV_tumor_-SUV_liver_.

### Small interfering RNA (siRNA) transfection

Cells were transiently transfected with siRNAs specific for beclin-1 (Qiagen, GS8678) or ATG5 (Qiagen, GS9474) in Lipofectamine RNAiMAX transfection reagent (Invitrogen, 13,778–150) according to the manufacturer’s protocol. After 48 h, the transfected cells were trypsinized and seeded into 96-well plates for drug treatment and cell viability analyses as described in previous sections.

### Autophagic flux assay with an GFP-LC3II reporter

NPC cells were infected with commercially available viruses engineered to carry the autophagy tandem sensor GFP-LC3II (Invitrogen, P36239) according to the manufacturer’s protocol. The cells were then transferred into a Vision 96-well plate (4titude, 4ti-0221) for live cell imaging. A LSM880 confocal microscope (Carl Zeiss) was used to obtain images of the GFP-labeled autophagosomes.

### qPCR analysis

Extraction of total RNA and reverse transcription to cDNA were performed using TRIzol® reagent (Invitrogen) and SuperScript® First-Strand Synthesis System for qPCR (Invitrogen), respectively, according to the manufacturer’s protocols. Expression levels of cancer stemness related genes were examined by qPCR. The primers and probes for different genes were designed using Universal Probe Library System (Invitrogen) as follow: SOX2 (probes#2; F: AATGCCTTCATGGTGTGGTC; R: CGTCTCCGACAAAAGTTTCC), NANOG (probes:#1; F: CCCCAGCCTTTACTCTTCCT; R: ACTGGATGTTCTGGGTCTGG), ABCG2 (probes:#2; F: GCTGCCAAGTTTCCTCTCTC; R: CCACGCCTACTAAACAGACGA), MMP2 (probes:#1; F: CAGGAGGAGAAGGCTGTGTT; R: GGTCAGTGGCTTGGGGTA), MMP9 (probes:#6; F: GAACCAATCTCACCGACAGG; R: GCCACCCGAGTGTAACCATA), GAPDH (probes:#60; F: AGCCACATCGCTCAGACAC; R: GCCCAATACGACCAAATCC), BMI1 (probes#1; F:AAAGACAAAGAGAAATCTAAGGAGGA; R: AACTTTCTTAAGTGCATCACAGTCA).

### Statistical analysis

The results are presented as mean values ± standard errors of the means. Comparisons between groups were performed using the two-tailed Student’s *t*-test with an assumption of equal variance. Statistical analyses were performed using Prism (GraphPad, Inc.). A *p*-value of less than 0.05 was considered to indicate statistical significance.

## Results

### Palbociclib inhibited cell cycle progression in NPC cell lines

**Fig. 1:**
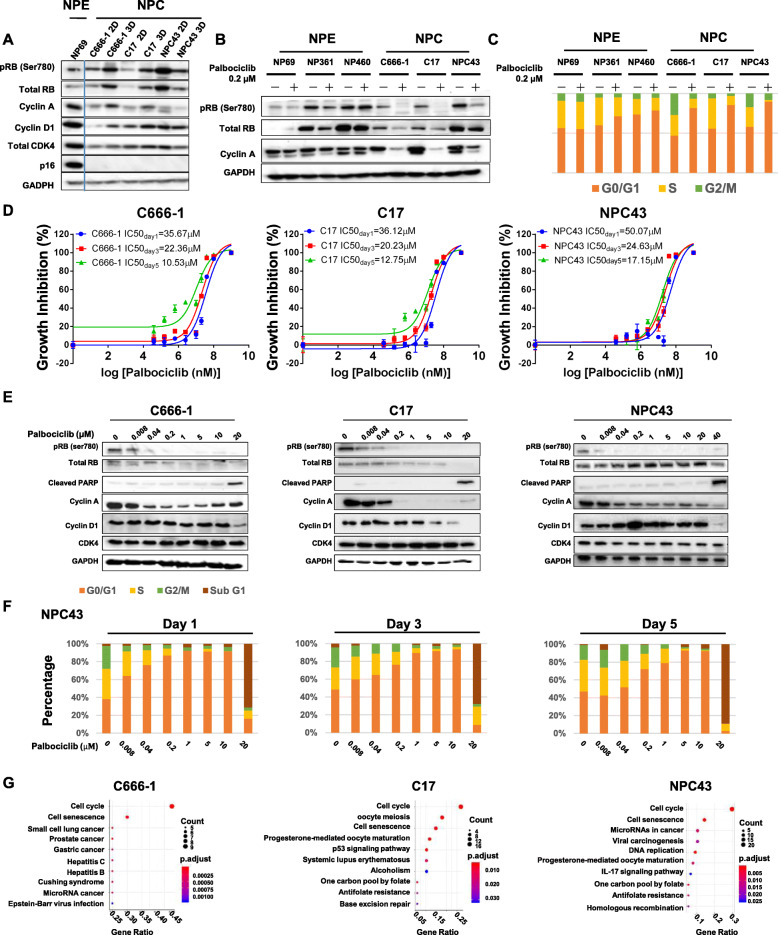
Palbociclib specifically inhibited nasopharyngeal carcinoma (NPC) cell growth in vitro by inhibiting cell cycle progression. **a**. Western blot analysis of cell cycle-related proteins in two- and three-dimensional cultures of NPC cells (C666–1, NPC43, and C17) and two-dimensional culture of nasophayngeal epithelial cells (NP69). The levels of markers predictive of palbociclib sensitivity were examined. **b**. Western blot analysis of total RB, phosphorylated (p) RB (Ser780), and cyclin A in NPC and NPE cells after treatment with 0.2 μM palbociclib for 24 h. **c**. Cell cycle distribution analysis of NPC and NPE cells after treatment with 0.2 μM palbociclib for 24 h. A prominent accumulation of cells in the G1 phase was observed in the NPC but not the NPE cell cultures after palbociclib treatment. **d**. IC_50_ values of palbociclib in NPC cell lines were determined at Days 1, 3 and 5 using a resazurin assay (**e**). Western blot analysis of cell cycle-related proteins [total RB, pRB (Ser780), cyclin A, cyclin D1, CDK4] and an apoptosis marker (cleaved PARP) in NPC cell lines after treatment with palbociclib at different doses for 24 h. Quantitative protein expression data are presented in Supplementary Figure S2. **f**. Cell cycle distribution analysis of NPC43 cells after treatment with different doses of palbociclib for 1, 3 and 5 days. **g**. RNA transcriptome analysis of the three NPC cell lines after treatment with palbociclib for 24 h. Differentially expressed genes (DEG) were subjected to a KEGG pathway analysis. The top 20 KEGG pathways with the most significant gene ratios are plotted in the order of gene ratio. The sizes of the dots are scaled according to the numbers of significant genes revealed by the DEG analysis. The colors of the dots represent the p-adjusted significance values. Genes involved in cell cycle regulation were significantly enriched in all three cell lines after treatment with palbociclib

Previous studies have established p16 inactivation, cyclin D1 overexpression, and functional RB as predictors of palbociclib sensitivity in cancer cells [22–25]. We first examined the levels of p16, cyclin D1, phospho-RB-Ser780 (an indicator of the functional status) and other relevant proteins in lysates of NPC cell lines and immortalized NPE cell line grown in both 2D monolayer and 3D spheroid cultures for 3 days (Fig. 1a). Notably, p16 protein was detected in the immortalized NPE cell line (NP69) but not the NPC cell lines (C666–1, C17 and NPC43). RB (Ser780) phosphorylation was detected in all the NPE and NPC cell lines, albeit at variable levels, and was closely associated with the expression of cyclin A, an indicator of the proliferation status. Cyclin D1 and CDK4 were universally detected in all the NPE and NPC cell lines (Fig. 1a).

We next examined whether 2D-cultured NPC cell lines and non-malignant NPE cell lines would exhibit different levels of sensitivity to palbociclib. All cell lines were treated with 0.2-μM palbociclib for 24 h, and changes in the levels of pRB, total RB and cyclin A from before and after treatment were monitored. As shown in Fig. 1b, treatment with palbociclib strongly reduced the levels of pRB in all three NPC cell lines but not in the immortalized NPE cells. Furthermore, the levels of cyclin A, a marker of cell cycle entry, were reduced in all three NPC cell lines after treatment with palbociclib, whereas this drug had no significant effect on cyclin A protein expression in the three immortalized NPE cell lines. A flow cytometric cell cycle analysis verified that a 24-h treatment with 0.2-μM palbociclib induced significant G1 arrest in all three NPC cell lines but had no significant effect on cell cycle progression in the three immortalized NPE cell lines (Fig. 1c). The proportions of cells in G1 increased by approximately 0, 14, and 6% in the immortalized NPE cell lines NP69, NP361, and NP460, respectively, after palbociclib treatment, compared to dramatic increases of 81, 55, and 53% in the NPC lines C666–1, C17, and NPC43, respectively, after treatment. These results suggest that NPC cells are more susceptible than NPE cells to the inhibitory effects of palbociclib.

### Low-dose and high-dose palbociclib exhibited cytostatic and cytotoxic effects, respectively, in NPC cell lines

We then evaluated the responses of the three NPC cell lines in response to various palbociclib concentrations over different time periods. The dose–response curves of C666–1, C17, and NPC43 are shown in Fig. 1d. The IC_50_ values of palbociclib in NPC43 at Days 1, 3, and 5 of treatment (50.07, 24.63, and 17.15 μM, respectively) were generally higher than those of C666–1 (35.67, 22.36, and 10.53 μM, respectively) and C17 cells (36.12, 20.23, and 12.75 μM, respectively). We further compared these IC_50_ values to the reported IC_50_ values of palbociclib in other cancer cell lines (adapted from the Genomics of Drug Sensitivity in Cancer databank, Supplementary Fig. S1) [33]. A mean IC_50_ of 35.7 μM on Day 3 of treatment was calculated for 770 cancer cell lines. Twenty-six of these cell lines were head-and-neck cancer cell lines that had a mean IC_50_ of 49.2 μM on Day 3, suggesting that our tested NPC cell lines may be more sensitive to palbociclib treatment than other head and neck cancer cell lines.

We also observed that palbociclib induced cytostatic effects in NPC cells at low doses (8 nM–5 μM) and cytotoxic effects at high doses (20–40 μM) (Fig. 1e). Cleaved-PARP, an apoptosis marker, was only detected in NPC cells treated with high-dose palbociclib (20, 20, and 40 μM for C666–1, C17, and NPC43, respectively) (Fig. 1e, Supplementary Fig. S2). However, we observed potent suppression of cyclin A protein expression and RB-Ser780 phosphorylation in cells treated with much lower doses of palbociclib. We also examined the growth inhibitory effect of palbociclib in 3D cultures of C666–1 and C17 (Supplementary Fig. S3) and confirmed the suppression of cyclin A expression and RB-Ser780 phosphorylation in these NPC spheroids.

We further assessed the cell cycle distribution of NPC43 cells exposed to different concentrations of palbociclib from Days 1 to 5 (Fig. 1f). A sub-G1 peak indicative of cellular apoptosis was only observed in NPC cells treated with high-dose palbociclib (20 μM). However, G1 arrest was observed even at lower doses of palbociclib, which further supports the distinct dose-dependent effects of palbociclib on NPC cells.

We then subjected the three NPC cell lines to RNA sequencing to identify the changes in gene expression in response to palbociclib treatment for 24 h. The differentially expressed genes were subjected to a KEGG database analysis (Fig. 1g), which revealed the downregulated expression of genes related to cell cycle progression in all three tested cell lines (Table 1). The RNA expression profiling analysis further confirmed that palbociclib induces cell-cycle arrest in NPC cells.

**Table 1:**
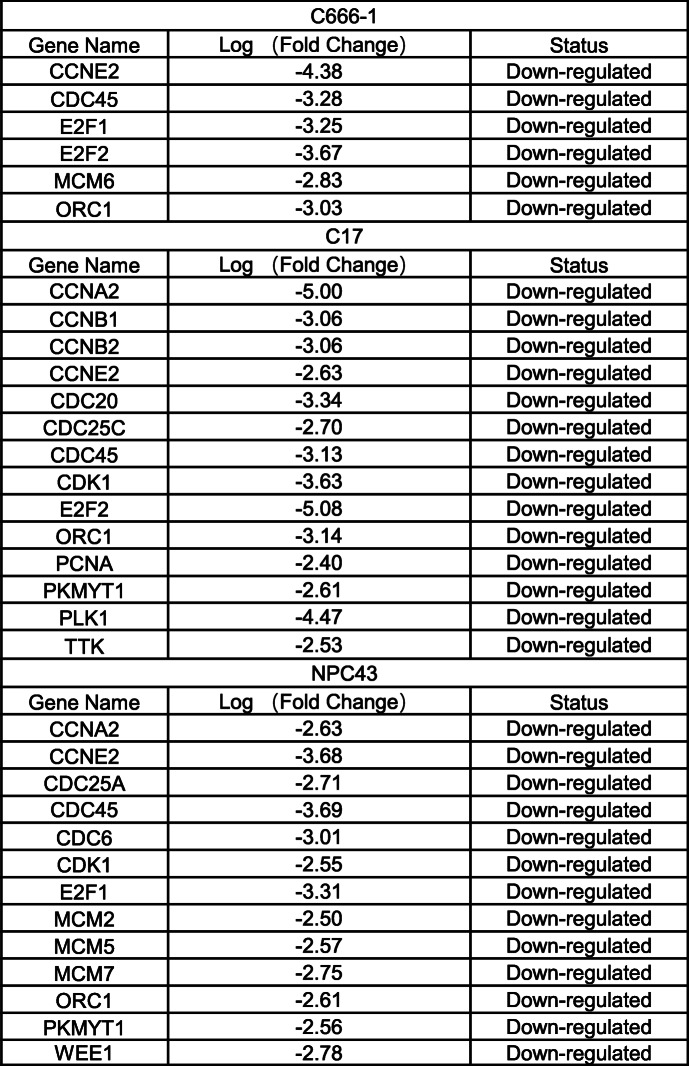
Downregulated cell cycle related genes in C666–1, C17 and NPC43 after 24 h treatment with 15 μM of palbociclib

### Oral administration of palbociclib suppressed the growth of multiple NPC xenograft models in vivo

This study included a comprehensive evaluation of the efficacy of palbociclib in six NPC preclinical xenografts models representing early- and advanced-stage NPC. Xeno32, Xeno76, and C666–1 xenografts were established from primary NPCs, Xeno23 and NPC43 were established from recurrent NPC and C17 was established from a metastatic NPC. Immunohistochemical analysis was used to evaluate the expression of p16, RB, and cyclin D1 proteins in these xenografts grown in NOD/SCID mice (Supplementary Fig. S4). Notably, all six xenografts lacked p16 protein but expressed readily detectable RB and cyclin D1.

**Fig. 2:**
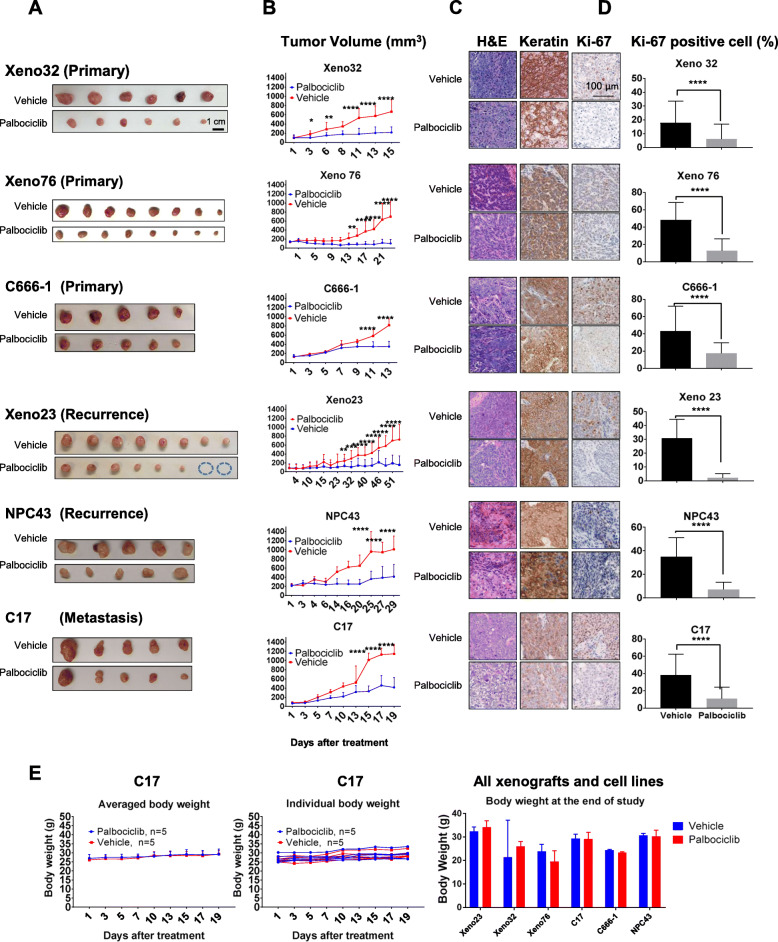
Palbociclib-mediated inhibition of nasopharyngeal carcinoma (NPC) xenograft *growth* in vivo in NOD-SCID mice. Different NPC xenograft models were treated with palbociclib at a dose of 75 mg/kg/day via oral gavage. **a-d**. Left panel: Excised subcutaneous tumors at the end of palbociclib treatment. Middle left panel: NPC tumor growth curves during palbociclib treatment, which was initiated when the subcutaneously injected or implanted NPC xenografts became palpable (100–200 mm^3^). The tumor size was calculated as the Length × Width × Height/2. Middle right panel: Hematoxylin-eosin staining and immunohistochemistry (IHC) analyses of NPC tissue slides. Right Panel: Statistical analysis of Ki-67-stained cells in the tumor sections. Represents a tumor that regressed completely after drug treatment. Enlarged images of tissues subjected to IHC are presented in Supplementary Figure S6. **e**. Left panel: Average body weights of C17 tumor-bearing mice were measured throughout the experiment. Middle panel: Individual body weights of C17 tumor-bearing mice. Right panel: Bar chart of average body weights in the treatment and vehicle groups of all NPC xenografted models. *P* < 0.0001 ****, *p* < 0.001***, *p* < 0.005**, *p* < 0.01*

For the treatment experiment, palbociclib was suspended in deionized water and administered daily (75 mg/kg/day) to mice bearing the Xeno32, Xeno76, C666–1, Xeno23, NPC43, and C17 xenografts for 15, 23, 13, 54, 29, and 19 days, respectively. The treatment duration in each group was dependent on the growth rate of each type of xenograft. Mice in the vehicle groups received an equivalent volume of deionized water on the treatment days. The tumor volumes were measured thrice weekly using a digital Vernier caliper. Mice were euthanized at the end of the treatment period, which was determined when the tumor diameter in the control group reached approximately 1 cm. As shown in Fig. 2a–b & Supplementary Fig. S5, oral palbociclib administration successfully inhibited the tumorigenic growth of all the NPC xenografts, and this finding supports the potential efficacy of palbociclib for the treatment of NPC.

We also performed immunohistochemical analysis to examine the expression of Ki-67, a commonly used marker of cancer cell proliferation, in NPC xenografts from the control and treatment groups at the end of the study (Fig. 2c–d). The method used to quantify Ki-67 staining in the sectioned tissues is illustrated in Supplementary Fig. S7. For all six NPC xenograft types, the percentages of Ki-67-expressing cells were significantly lower in the palbociclib treatment groups than in the control groups. The body weights of treated and control animals were measured throughout the treatment period, and no significant differences were observed. These results suggest that the palbociclib dosages administered to the NPC-bearing mice had no major adverse effects on general well-being (Fig. 2e).

The C666–1 cells used in this study could colonize the lungs of mice after tail vein injection. We therefore examined whether palbociclib could suppress the metastasis of C666–1 cells to the lungs in vivo. Four NOD/SCID mice each were included in the palbociclib treatment and control groups, and the mice were treated for 124 days. At the end of the study, lung tissues were dissected from the mice, fixed, and processed to examine the growth of C666–1 cells. Histological analysis of hematoxylin–eosin-stained lung tissues revealed that palbociclib effectively suppressed the colonization of C666–1 cells (Supplementary Fig. S8).

### Combination treatment with palbociclib and other drugs revealed antagonistic effects with cisplatin but synergistic effects with SAHA against NPC cells

**Fig. 3:**
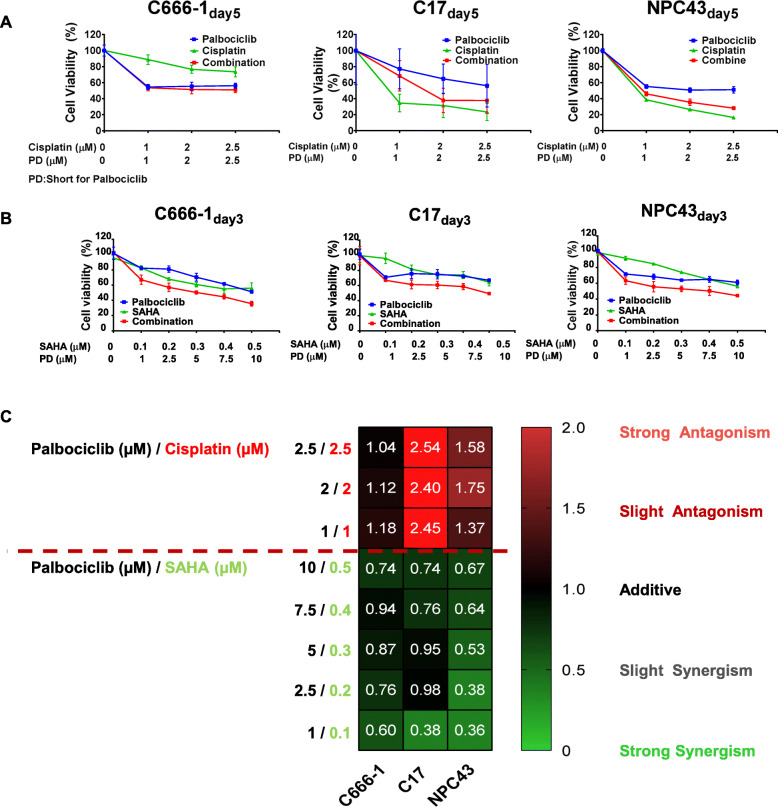
Palbociclib suppressed the cytotoxicity of cisplatin but potentiated the growth inhibitory effect of SAHA in NPC cells. **a**. Combined palbociclib + cisplatin treatment did not enhance growth suppression in NPC cells (NPC43 and C17) relative to treatment with cisplatin treatment alone. **b**. Combined treatment with palbociclib + SAHA strongly suppressed viability in the C666–1, C17 and NPC43 cell lines. **c**. Combination index (CI) values were calculated using the Chou–Talalay Method to demonstrate the antagonistic, additive, and synergistic effects of drug combinations on the viability of NPC cells. The CI values of palbociclib + cisplatin and palbociclib + SAHA at various doses in the three NPC cell lines are presented as a heatmap. CI values > 1 were observed in all cell lines treated with palbociclib + cisplatin, suggesting an antagonistic effect between these drugs. CI values < 1 were observed in all NPC cell lines treated with palbociclib + SAHA, suggesting a synergistic effect between these drugs

The treatment outcomes of patients may be worsened by drug resistance and the adverse effects of high-dose therapies. Combination treatment with two or more anticancer drugs that target different cellular pathways is a common strategy used to minimize treatment toxicity. We therefore examined the effects of combination treatments of palbociclib and other therapeutic agents, namely cisplatin or SAHA, on NPC cells. Cisplatin is commonly used for the clinical management of NPC, and SAHA has been evaluated in preclinical models of NPC [34]. These drugs have distinct mechanisms of action: cisplatin induces DNA damage, whereas SAHA inhibits HDAC activity. To determine whether the combined use of palbociclib and cisplatin or SAHA would have antagonistic, synergistic or additive effects, NPC cells were treated in vitro with either palbociclib monotherapy or a combination of palbociclib with cisplatin or SAHA at serially concentrations. In all three tested NPC cell lines, the combined use of palbociclib plus cisplatin did not further suppress NPC cell growth. Rather, this combination did not act as effective as either cisplatin or palbociclib monotherapy in suppressing NPC growth (Fig. 3a). This antagonistic interaction was not unexpected because cisplatin induces DNA damage, which could only be elicited once the cell entered the cell cycle. The inhibitory effects of palbociclib on cell cycle entry could protect cells from the cytotoxic effect of cisplatin. In contrast, we observed that treatment with SAHA significantly enhanced the ability of palbociclib to suppress NPC cell growth (Fig. 3b).

We also used the Chou–Talalay method to calculate the combination index (CI) of each drug combination as an indicator of the potential synergistic, additive, or antagonistic effects of the two reagents [35, 36]. CI values of < 1, 1, and > 1 imply synergistic, additive, and antagonistic effects, respectively [35, 36]. The combination of palbociclib and cisplatin at concentrations of 2.5 μM each yielded CI values of 1.58, 2.54, and 1.041 for NPC43, C17, and C666–1, respectively, indicating a significant antagonistic effect of this combination in NPC cell lines (Fig. 3c). In contrast, the Chou–Talalay calculation confirmed the synergistic effect of the combination of palbociclib with SAHA at respective concentrations of 1.0 and 0.1 μM, which yielded CI values of 0.36, 0.38, and 0.6 in NPC43, C17, and C6661 cells, respectively (Fig. 3c).

### Combined treatment with palbociclib and SAHA suppressed NPC xenograft growth in vivo

**Fig. 4:**
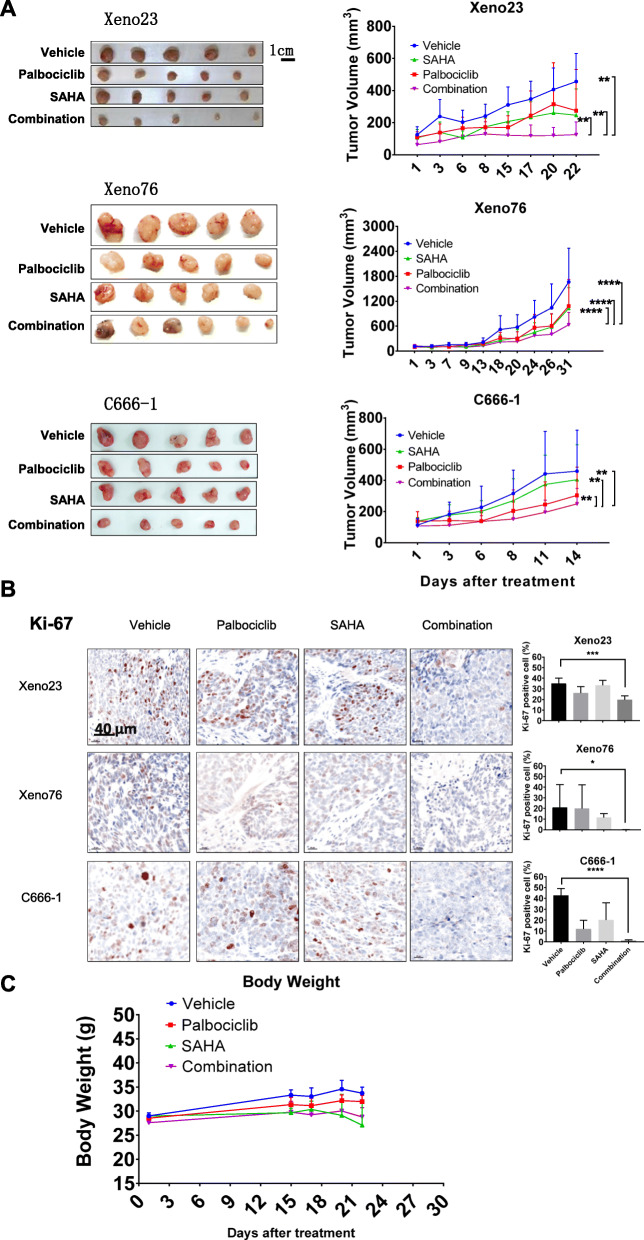
Combined treatment with palbociclib and SAHA more strongly suppressed the in vivo growth of NPC tumors than either monotherapy. **A.** Mice bearing Xeno23, Xeno76, and C666–1 xenografts were treated with the vehicle, palbociclib (75 mg/kg every other day), SAHA (20 mg/kg every other day) or palbociclib + SAHA. The tumor sizes were compared between groups using Student’s t-test (*p* < 0.0001 ****, *p* < 0.001***, *p* < 0.005**, *p* < 0.01*). **b**. Representative photos of tumor sections from Xeno23, Xeno76, and C666–1 xenografts in control and treatment groups after IHC for Ki-67. The percentage of Ki-67–positive cells was significantly lower in tumors subjected to combination treatment. The statistical analysis was calculated based on five randomly selected areas of each treated tumor section. Enlarged images of tissue sections subjected to IHC are presented in Supplementary Figure S9. **c**. Average body weights of Xeno23 tumor-bearing mice during the treatment period

Based on the results of our Chou–Talalay analysis [35, 36], we aimed to confirm the synergistic suppressive effect of combined treatment with palbociclib and SAHA on the growth of Xeno23, Xeno76, and C666–1 tumors in vivo. NPC xenograft-bearing mice were treated with palbociclib (75 mg/kg on alternate days) and/or SAHA (20 mg/kg on alternate days). This reduction in the palbociclib dose from a monotherapeutic dose of 75 mg/kg per day to the same dose on alternating days in the combination treatment was done to ensure that any potential additive or synergistic effect of SAHA could be observed. The average tumor volumes, growth curves, and histological appearances of all NPC xenografts subjected to monotherapies and combination therapies are shown in Fig. 4a. Significant tumor growth inhibition was observed in all mice in the combination palbociclib and SAHA treatment group, compared to both monotherapy groups, and the decreased tumor volume was particularly prominent in Xeno23 and C666–1 xenograft models. We further examined Ki-67 expression in NPC cells in the control and treatment groups as a measure of the cell proliferation status. In all three NPC xenograft models, all combination treatment groups exhibited significant decreases in Ki-67 expression relative to the control groups (Fig. 4b & Supplementary Fig. S9).

We subjected Xeno23 model mice to ^18^F-FDG microPET scans to examine the metabolic rates in the tumors after combination treatment, which were represented by the mean SUV (SUV_mean_). After normalization to the liver basic metabolic rate, the tumor metabolic rates were 0.76 and 0.09 in the vehicle and combination treatment groups, respectively (Supplementary Fig. S10). The body weights of mice in both groups were also measured throughout the treatment period, and no significant between-group differences were observed (Fig. 4c).

### Autophagy-associated cell death as a mediator of enhanced cytotoxicity induced by combined treatment in NPC cells

We sought to explore some of the mechanisms underlying the enhanced death of NPC43 cells in response to palbociclib and SAHA monotherapy or combination therapy (Supplementary Fig. S11). The levels of multiple protein markers associated with the cell cycle*,* differentiation and apoptosis were analyzed by Western blotting. Palbociclib monotherapy inhibited cell cycle progression in treated NPC43 cells, as shown by the suppression of RB-Ser780 phosphorylation and cyclin A expression. SAHA monotherapy at the indicated doses was a much less effective suppressor of RB phosphorylation and cyclin A expression, which highlights the different modes of action of these drugs. We did not observe significant effects of palbociclib and SAHA monotherapy or combination therapy on involucrin (a squamous cell differentiation marker), cleaved-PARP, or cleaved-caspase 3 (apoptosis markers) in NPC43 cells treated with SAHA alone or in combination with palbociclib.

**Fig. 5:**
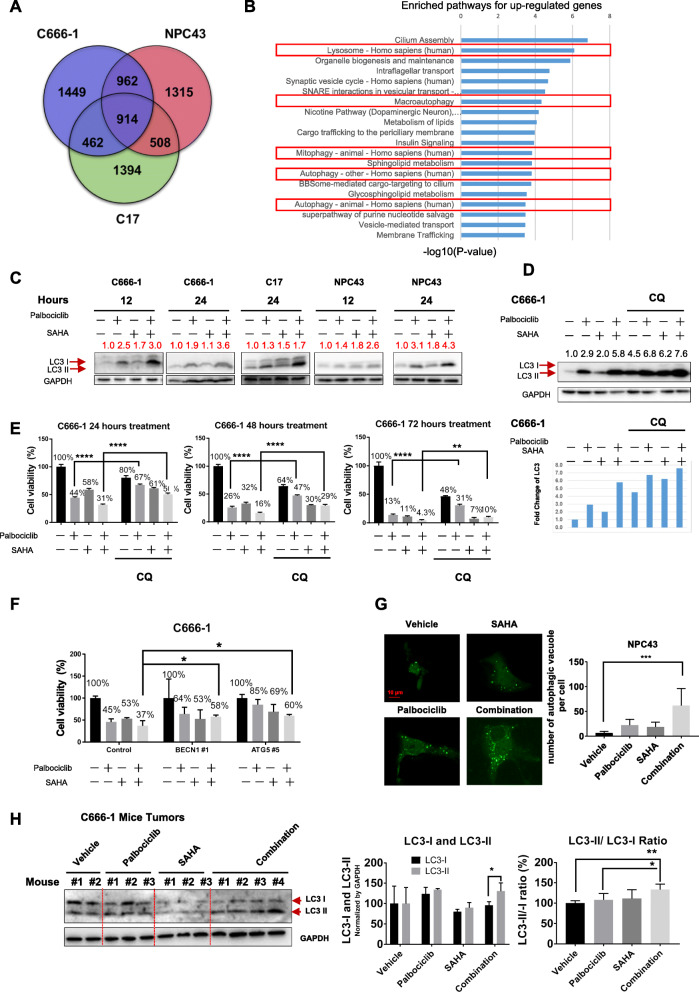
Involvement of autophagy-related pathways in the enhanced cell death of NPC cells under combined treatment with palbociclib and SAHA. **a**. Venn diagram of the numbers of upregulated DEGs in the three NPC cell lines after combination treatment with palbociclib + SAHA. The number in the central region denotes the number of overlapping genes in all three cell lines. A total of 914 genes were commonly upregulated in all three cell lines treated with palbociclib + SAHA relative to their vehicle-treated counterparts. **b**. The top 20 enriched pathways among the 914 DEGs were identified from the CPBD database and are listed in the order of *p*-value, which depended on the numbers and fold changes of the genes. Upregulated genes were enriched in autophagy or its related cellular functions (highlighted in red rectangles). **c**. Detection of the autophagy marker, LC-3II, in the C666–1, C17, and NPC43 cell lines treated with the vehicle, palbociclib (15 μM), SAHA (5 μM), or palbociclib + SAHA. The numbers above the blots indicate the normalized grayscale values of LC3-II. **d**. Autophagic flux analysis of C666–1 cells treated with palbociclib, SAHA, or palbociclib + SAHA and chloroquine (CQ), an inhibitor of autophagic flux (25 μM), for 24 h. Lower panel: Bar chart demonstrating the fold changes in LC3-II relative to the vehicle control. **e**. Viability of C666–1 cells subjected to the same treatment as in Fig. 5d for 24, 48, and 72 h. **f**. Knockdown of the autophagy-related proteins BECN1 and ATG5 via siRNAs partially reversed the cytotoxic effects in C666–1 cells treated with palbociclib and SAHA for 24 h. Cell viability was tested using the resazurin assay. Corresponding verification of the siRNA-mediated knockdown efficiency is presented in Supplementary Figure S14. **g**. Detection of the autophagy level in NPC43 cells using the Autophagy Tandem Sensor GFP-LC3B kit. The cells were treated with palbociclib and SAHA for 24 h as described in Fig. 5c. The presence of GFP-LC3-II–positive autophagic vacuoles in the NPC cells was visualized using a Carl Zeiss LSM 880 confocal microscope. **h**. Combined treatment upregulated the turnover of LC-3II in C666–1 the tumors in vivo relative to the other treatment groups. Left panel: Western blot analysis of LC-3I and LC-3II in tumors excised from mice in different treatment groups. Middle panel: Bar chart of the LC3-I and LC3-II levels relative to the corresponding GAPDH level in each treatment group, as detected in the left panel. Right panel: Bar chart of the LC3-II/LC3-I ratio in each treatment group as detected in the left panel. *P* < 0.0001 ****, *p* < 0.001***, *p* < 0.005**, *p* < 0.01*

Reactivation of the EBV lytic cycle was reported to play a role in NPC cell apoptosis after drug treatment [34]. Although we detected the lytic gene, *BZLF1*, at the single-cell level using RNAscope (Supplementary Fig. S12), less than 1% of the tumor cells expressed *BZLF1* before or after the treatment, suggesting that EBV reactivation did not account for the enhanced cell death observed in response to combined treatment. Therefore, to explore the biological pathways that may have contributed to the enhanced cell death associated with combined palbociclib and SAHA treatment, we compared the RNA-sequencing profiles of three NPC cell lines (C666–1, NPC43, and C17) to identify differential gene expression (DEG) after palbociclib and SAHA treatment. The resulting Venn diagram revealed 914 upregulated genes shared by all three NPC cell lines after treatment with both drugs (Fig. 5a). A KEGG pathway analysis of these upregulated genes was conducted to identify the top 20 pathways based on the gene ratios related to each pathway. Interestingly, many of the upregulated genes were involved in autophagy pathways, as listed according to the CPDB (ConsensusPathDB) database (Fig. 5b). The autophagy-related genes involved in the enriched lysosome, macroautophagy, mitophagy, and autophagy pathways are listed in Table 2. We performed Western blot analysis of NPC cells treated with a combination of palbociclib and SAHA and identified increases in the LC3-II (the phosphatidylethanolamine conjugated form of LC3), which indicated increases in the cellular autophagy flux (Fig. 5c).

**Table 2:**
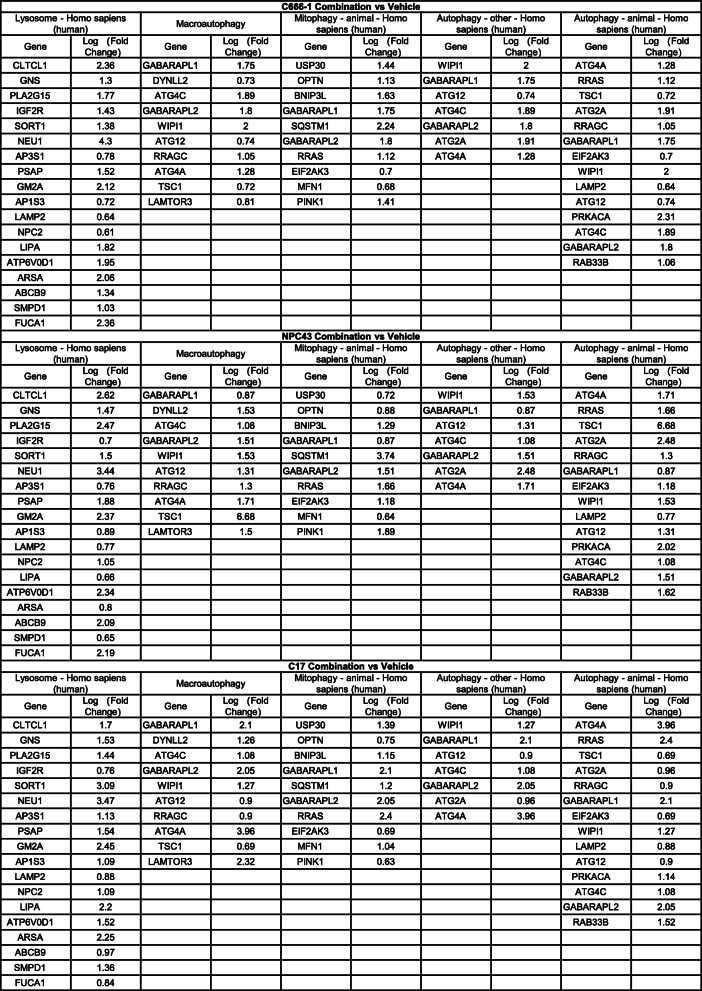
Upregulation of autophagy-related genes of C666–1, NPC43 and C17 under the combined treatment of palbociclib and SAHA compared to vehicle treatment

Elevated LC3-II may be attributable to enhanced autophagosome formation or blocked autophagic degradation. We next evaluated the effect of chloroquine (CQ), a specific inhibitor that blocks autophagic flux by decreasing autophagosome–lysosome fusion, on the LC3-II and viabilities of NPC cell lines. In a fully autophagic cell, CQ would induce an increased accumulation of LC3-II. If autophagy is blocked at the autophagic degradation step, however, CQ treatment would not further increase the level of LC3-II [37]. As shown in Fig. 5d, we determined a higher level of LC3-II in C666–1 cells subjected to combination treatment (~ 5.8-fold increase after normalization to GAPDH expression) than in cells subjected to palbociclib (~ 2.9-fold increase) or SAHA monotherapy (~ 2-fold increase), indicating that combination therapy induced an increase in autophagic flux. In the presence of CQ, the LC3-II level increased by 7.6-fold in C666–1 cells treated with the combination of palbociclib and SAHA, compared to the control. Importantly, treatment with CQ also led to a significant increase in viability in cells treated with the combination therapy, compared to palbociclib monotherapy (50% vs. 31%) at 24 h (Fig. 5e), and similar trends were observed at 48 and 72 h and in C17 and NPC43 cells (Supplementary Fig. S13).

We further determined that knockdown of the autophagy-related proteins ATG5 and beclin-1 also reversed the NPC cell death induced by palbociclib monotherapy or palbociclib and SAHA combination treatment (Fig. 5f, Supplementary Fig. S14). We further visualized autophagosomes in cells transfected to express GFP-LC3-II and determined that these structures were enriched in cells treated with palbociclib alone or in combination with SAHA (Fig. 5g, Supplementary Fig. S15).

We also examined the levels of LC3-II in proteins extracted from C666–1 xenograft tumors from mice treated with a combination of palbociclib and SAHA. We observed a significant increase in the LC3-II/LC3-I turnover ratio in the combination treatment group relative to the monotherapy groups, consistent with the results of the Western blot analysis of cultured C666–1 cells (Fig. 5h). Taken together, our results suggest that autophagy plays a role in the cytotoxicity induced by combined treatment with palbociclib and SAHA.

### Sensitivity of palbociclib-resistant NPC cells to cisplatin treatment

**Fig. 6:**
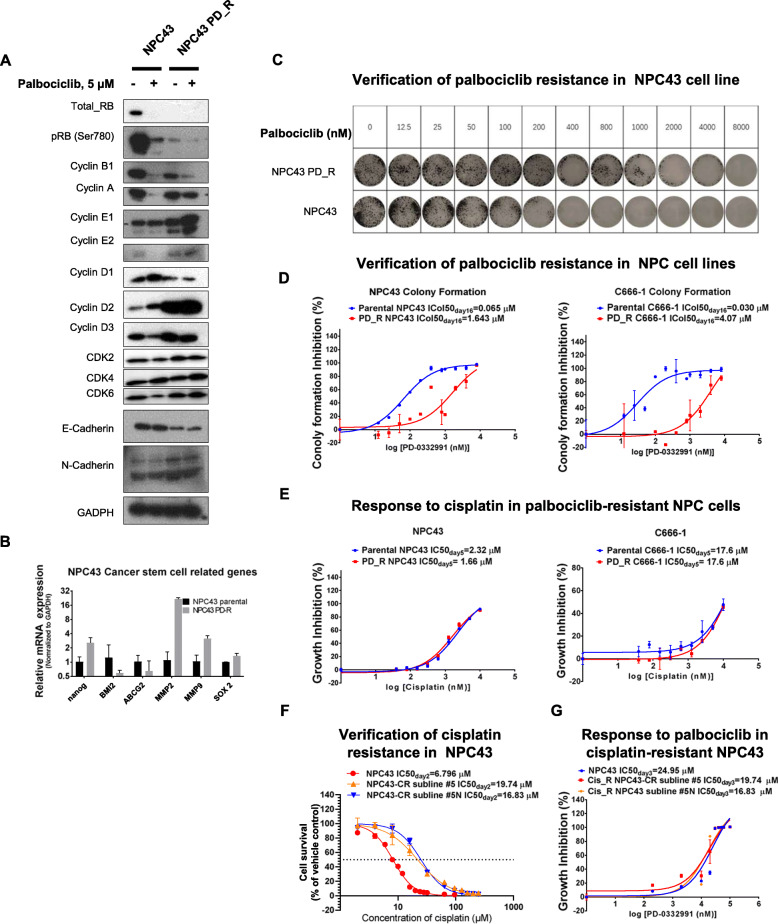
Palbociclib-resistant and cisplatin-resistant NPC cells remain sensitive to cisplatin and palbociclib, respectively. **a**. Western blot analysis of the cell cycle-related genes E-cadherin and N-cadherin in parental and PD-resistant NPC43 cell lines in the presence or absence of palbociclib treatment (5 μM). **b**. The cancer stem cell–related gene expression profiles in parental and palbociclib-resistant (PD_R) cells were evaluated by qPCR. **c**. A colony formation assay verified the resistance of NPC43 PD_R cells to palbociclib. **d**. Determination of the concentrations that would induce a 50% inhibition of colony formation (ICol_50_) in both the parental and pablbociclib-resistant NPC43 and C666–1 cell lines. **e**. The sensitivity of NPC43 PD_R and C666–1 PD_R cells to cisplatin was compared to that of the respective parental cell lines through a resazurin cell viability assay. **f**. Cisplatin resistance in NPC43 sublines #5 and #5 N was verified through a resazurin cell viability assay. **g**. The sensitivity of parental and cisplatin-resistant NPC43 cells to palbociclib was tested through a resazurin cell viability assay

Cancer cells often develop mechanisms to resist the inhibitory effects of a particular drug after prolonged treatment. Consequently, NPC cells may eventually gain resistance to palbociclib. We conducted a preclinical investigation to determine whether palbociclib-resistant NPC cells would retain their responsiveness to other chemotherapeutic agents, such as cisplatin. To establish palbociclib-resistant (PD_R) NPC cell lines, we treated C666–1 and NPC43 cells with increasing doses of palbociclib over a period of 1.5 years. We then characterized the levels of cell cycle-related proteins in parental and resistant NPC43 cells in response to treatment with palbociclib or vehicle (Fig. 6a). The levels of RB and phospho-RB (Ser780) were decreased in NPC43 PD_R cells. Notably, resistant cells maintained the basal expression of cyclin A even when treated with 5 μM palbociclib, which was shown to inhibit cyclin A expression in parental NPC43 cells. NPC43 PD_R cells also expressed higher levels of cyclin E1, cyclin D2, and cyclin D3. Taken together, our results suggest that the resistant cells may have acquired alternate CDK4/6/cyclin D1/RB-independent pathways to maintain cell proliferation in the presence of palbociclib. The resistant lines also exhibited downregulated E-cadherin expression and upregulated N-cadherin expression, suggesting that these cells may be more prone to metastasis. A qPCR analysis also revealed elevated expression of the cancer stemness-related genes *MMP2*, *MMP9*, *Nanog* and *SOX2* (Fig. 6b), suggesting that NPC43 PD_R cells may have acquired an increase in cancer stemness.

We conducted a colony formation assay to verify the palbociclib-resistant status of NPC43 PD_R cells (Fig. 6c). Parental NPC43 cells could no longer sustain colony formation under treatment with 0.8 μM palbociclib, whereas NPC43 PD_R cells could maintain colony formation even under treatment with a 10-fold higher concentration of palbociclib (8 μM). We also established a C666–1 PD_R cell line and determined the concentrations that would induce a 50% inhibition of colony formation (ICol_50_) in both the parental and PD_R NPC43 and C666–1 cell lines. Relative to their parental lines, NPC43 PD_R and C666–1 PD_R exhibited increases in the ICol_50day16_ values of 25-fold (from 0.065 to 1.645 μM) and 133.5-fold (from 0.03048 to 4.07 μM), respectively (Fig. 6d).

We next examined the responses of the two palbociclib-resistant NPC cell lines to treatment with cisplatin (Fig. 6e) and observed comparable IC_50day5_ values in both the parental and PD_R NPC43 and C666–1 lines. This observation suggests that palbociclib-resistant cells retain sensitivity to cisplatin, and therefore cisplatin could potentially be used to treat patients with palbociclib resistance.

### Sensitivity of cisplatin-resistant NPC cells to palbociclib treatment

Platinum-based therapies are used as a first-line treatment for primary NPC, a salvage treatment for recurrent disease and a palliative treatment for metastasis [2, 3, 38]. Therefore, we assessed whether cisplatin-resistant NPC cells would remain responsive to palbociclib treatment. In our analysis, two cisplatin-resistant sublines of NPC43 exhibited approximately 6-fold increases in the IC_50_ values relative to the parental NPC43 (IC_50day2_ increases from 6.8 to 48.58 and 51.31 μM; Fig. 6f). We then determined the efficacy of palbociclib in these cisplatin-resistant sublines and observed IC_50day3_ values of 28.61 μM versus 19.74 μM and 16.83 μM for the NPC43 parental versus the cisplatin-resistant sublines (Fig. 6g). This slight decrease in the IC_50_ indicated that the cisplatin-resistant NPC cells retained the parental sensitivity to palbociclib.

## Discussion

In this study, we aimed to generate preclinical evidence to support the administration of palbociclib as a targeted drug therapy for NPC and to assess the compatibility of palbociclib with other chemotherapies for the treatment of this malignancy. Recent genomic profiling analyses of NPC determined the existence of few druggable targets in NPC [10, 11, 13, 14]. Nevertheless, dysregulated p16–CDK4/6–cyclinD1 signaling is a well-known common event in the pathogenesis of NPC that promotes uncontrolled tumor growth and metastasis.

Targeted therapies for various types of cancer have been widely adopted and continuously investigated over the past decade, and many FDA-approved targeted therapies have been developed based on the genomic properties of prevalent malignancies such as gastric, breast, and liver cancers. In contrast, concurrent and adjuvant chemotherapy remain the main lines of treatment for NPC, particularly in the advanced stage of disease [39]. Most current phase III clinical trials of NPC are assessing the therapeutic efficacies of combination of conventional cytotoxic drugs (e.g., cisplatin, doxorubicin, and 5-fluorouracil) [40]. Therefore, the development of effective and selective therapeutic agents with novel molecular targets may improve the efficacy of NPC treatment. Very few clinical trials are investigating the use of targeted drugs for the treatment of NPC. The clinical effects of camrelizumab and tislelizumab, which target programmed cell death-1 (PD-1), and of anlotinib and nimotuzumab, which respectively target receptor tyrosine kinases and epidermal growth factor receptor (EGFR) [41–44], remain to be reported.

In this study, we evaluated the tumor-suppressive effects of palbociclib in a large panel of NPC preclinical models, including newly established and well-characterized xenografts and cell lines derived from primary, recurrent, and metastatic NPCs [45]. We confirmed that functional RB and inactivated p16 are common features in our tested NPC models, as described in our previously published study [8]. We also confirmed the sensitivity of NPC to palbociclib both in vitro in NPC cell lines and in vivo in xenografts and determined that this drug induced cell cycle arrest in the G1 phase. All the examined NPC xenografts were sensitive to treatment with 75 mg/kg palbociclib each day, which is comparatively lower than the doses used in many reported in vivo studies of other cancer types (150–200 mg/kg/day) [46–48]. The variable responses of different NPC xenografts to palbociclib (Supplementary Fig. S5) may be related to the varied basal expression of some markers that are sensitive to palbociclib or to other intrinsic properties of various NPC cell lines and xenografts. In our study, the Xeno23, Xeno76, and Xeno32 xenografts exhibited the strongest responses to palbociclib; in these models, tumor growth was arrested throughout the treatment (Fig. 2b). Although the NPC xenografts from established NPC cell lines (NPC43, C666–1, and C17) exhibited slight increases in tumor size during the treatment periods, the tumors remained significantly smaller in the treatment groups than in the vehicle control groups.

Palbociclib is an FDA-approved drug for clinical use, so potential adverse effects should not be a significant issue. In this preclinical trial, we did not observe significant differences in body weights between the mice in the treatment and control groups. Interestingly, we also observed that palbociclib could inhibit the in vivo colonization of lung tissues with intravenously injected NPC cells, which suggests that this drug could potentially inhibit NPC metastasis (Supplementary Fig. S8). Consistent with this observation, palbociclib was shown to inhibit breast cancer metastasis in animal models through a mechanism that involves inhibition of the c-Jun/COX-2 signaling pathway [49]. However, the detailed molecular mechanism by which palbociclib suppressed NPC metastasis warrants further investigation.

The potent suppressive effects of palbociclib on the growth of NPC xenografts derived from patients with primary, recurrent, and metastatic tumors support the application of this drug in clinical trials related to NPC treatment. A previous preclinical study evaluated another CDK4/6 inhibitor, ribociclib, that targets a different site in the ATP-binding pocket of CDK4/6 and similarly demonstrated that the inhibition of this signaling pathway could inhibit the growth of NPC cells [50]. An integrated genomic and transcriptomic study of five patient-derived xenografts also determined that the copy numbers of *CCND1* and *CDKN2A* are potential targets of palbociclib and may mediate the suppression of tumor growth [51]. A case report also demonstrated the clinical benefits of palbociclib treatment in a patient with previously treated metastatic NPC with *CDK4* amplification [52]. Taken together, these observations emphasize the strong therapeutic value of targeting the dependency of NPC cells on the CDK4/6–cyclinD1 pathway.

In the second part of this study, we furthered our exploration of the efficacy of combination treatments that include palbociclib for the treatment of NPC. Combinations of chemotherapy drugs are commonly used in cancer treatment to prevent drug resistance, reduce drug dosages, and minimize adverse effects. Because platinum-based chemotherapy (e.g., cisplatin) is the most commonly used NPC treatment option, we first assessed the combined effect of cisplatin and palbociclib on the viability of NPC cells in vitro. Previous studies have reported antagonistic effects when CDK4/6 inhibitors are combined with other specific chemotherapeutic drugs [53, 54] such as taxane, PLK1 inhibitors, gemcitabine, and other drugs with mechanisms of action that rely on continuous cell cycle progression. In this study, palbociclib also protected NPC cells from the cytotoxic effects of cisplatin (Fig. 3a), possibly because palbociclib induces cell cycle arrest in the G1 phase. To mediate its cytotoxic actions, cisplatin induces DNA damage by crosslinking purine bases on DNA, interfering with DNA damage repair and triggering apoptosis and cell death [55]. These actions take place in the S phase of cell cycle and therefore may be abrogated by the effects of palbociclib.

In contrast, we observed a synergistic effect when palbociclib was combined with SAHA, an FDA-approved drug for the treatment of cutaneous T cell lymphoma [56]. SAHA is a broad-spectrum HDAC inhibitor that disables HDAC by removing acetyl groups from histone proteins, thus disrupting the regulation of gene expression [57, 58]. SAHA can induce growth arrest and death in a broad range of transformed cells both in vitro and in vivo at concentrations that induce few or no toxic effects on normal cells [26, 59]. Although the exact mechanisms remain to be elucidated, the anticancer effect of SAHA is attributed to dysregulation of the expression of genes involved in cell proliferation and death pathways [26]. In this study, we confirmed the synergistic effects of palbociclib and SAHA in three authenticated EBV-infected NPC cell lines (Fig. 3b). We further observed that the addition of SAHA enhanced the tumor inhibitory effect of palbociclib in our Xeno76, Xeno23, and C666–1 xenograft models (Fig. 4a). This is the first report to demonstrate the ability of SAHA to enhance the efficacy of palbociclib for the treatment of NPC.

We sought to understand the mechanisms that underlie enhanced NPC cell death in response to this combination treatment. In a previous study of NPC, SAHA was shown to induce apoptosis and suppress tumor growth by activating the lytic cycle of EBV [34, 60]. In our study, we demonstrated the lytic reactivation of EBV at the single-cell level by using an RNAscope analysis of the lytic gene, *BZLF1* (Supplementary Fig. S12). However, fewer than 1% of the xenograft tumor cells were shown to express this gene, suggesting that lytic reactivation was not the major cause of the enhanced cell death in response to combined treatment. We also did not detect increased levels of apoptosis and differentiation markers in cells subjected to the combined treatment (Supplementary Fig. S11), suggesting that these processes were not responsible for the enhanced cell death. Interestingly, RNA sequencing and Western blot analyses revealed the common induction of autophagy-related pathways in all three NPC cell lines in response to the combination of palbociclib and SAHA (Fig. 5). Evidence suggests that autophagy can participate in a caspase-independent form of programmed cell death induced by anticancer drugs [61]. Autophagic cell death is due to the accumulation of presumably toxic autophagic cargo in cells with a defective ability to degrade this material in lysosomes. Autophagy has been reported to play contradictory roles in tumor initiation and progression, and both autophagic repression and stimulation have been identified as therapeutic approaches, depending on the cellular context of the tumor.

Autophagy-associated cell death can be induced therapeutically by modulating regulators of autophagy. In a previous study, a combination of autophagy-associated mTOR inhibition and radiation yielded enhanced therapeutic effects in cancer cells and xenografted tumors [62]. In our study, palbociclib increased the expression of LC3-II, and the combined use of palbociclib and SAHA further augmented this expression (Fig. 5). The autophagy inhibitor CQ can alkalize the lysosomal lumen and block autolysosomal degradation. In our study, CQ treatment further enhanced the increased expression of LC3-II in groups treated with palbociclib monotherapy or combined palbociclib and SAHA, indicating that palbociclib itself can upregulate autophagic flux. Furthermore, we found that CQ could inhibit the death of C666–1 cells exposed to palbociclib alone or in combination with SAHA. In vivo, the combined treatment induced higher LC3-II/LC3-I ratios in the xenograft tumors (Fig. 5h). These observations suggest that autophagy is a factor in the cell death induced by palbociclib monotherapy or combined palbociclib and SAHA therapy. A previous study of hepatocellular carcinoma cells demonstrated that palbociclib induced autophagy in a CDK4/6-independent manner; in those cells, autophagy was induced via a mechanism involving 5′ AMP-activated protein kinase (AMPK) activation and protein phosphatase 5 (PP5) inhibition [63]. Other studies reported that HDAC inhibitors could induce caspase-independent autophagic cell death in HeLa and chondrosarcoma cells [64, 65]. In our NPC cell systems, SAHA appeared to potentiate the autophagy-inducing effect of palbociclib, which may have led to the massive degradation of essential cellular structures and autophagy-associated cell death. Investigations are needed to further elucidate the role of autophagy as a mediator of the enhanced cytotoxic effects of palbociclib, either alone or in combination with SAHA, especially in the context of NPC cells.

We have demonstrated the potent effect of palbociclib as a suppressor of the growth of primary, recurrent and metastatic cancer cells and anticipate a first-in-human clinical trial of this drug in patients with NPC. In 2009, 16 patients with treatment-naïve World Health Organization histological grade III, stage II (*n* = 2), III (*n* = 6), and IVB (*n* = 8) NPC were treated with the non-selective CDK inhibitor seliciclib [51]. However, the use of seliciclib in clinical trials was displaced by the subsequent development of specific inhibitors to CDK4/6 such as palbociclib. However, palbociclib use may eventually lead to drug resistance [66, 67]. Therefore, we examined whether palbociclib-resistant NPC cells would remain vulnerable to cisplatin treatment and observed the acquisition of a new cell cycle pathway independent of RB phosphorylation (Fig. 6a). Cyclin A expression, which indicates proliferation, could be maintained at a high level in these cells even under the treatment of palbociclib. Cyclin E1 overexpression was also observed in palbociclib-resistant NPC43 cells. A previous study identified E2F activation via the cyclin E–CDK2 axis as a factor that reverses the inhibition of CDK4/6 and enables cell cycle progression from the G1 to the S phase [67]. NPC cells may use a similar mechanism to develop resistance to palbociclib. Importantly, these palbociclib-resistant NPC cell lines remained sensitive to cisplatin (Fig. 6e), suggesting that this platinum-based drug could still be used to treat patients who develop resistance to palbociclib.

Despite the promising use of palbociclib as a therapeutic option for NPC, irradiation and concurrent cisplatin chemotherapy will remain the first-line treatment options for most primary cases until the benefits of palbociclib can be demonstrated in clinical trials of recurrent or metastatic NPC patients. Cisplatin is also used in salvage chemotherapeutic regimens for the treatment of recurrent tumors and even palliative regimens for the treatment of metastases, as approved targeted therapies for NPC are not available [3]. Accordingly, we sought to verify whether palbociclib would effectively target cisplatin-resistant NPC cell lines. In our study, palbociclib could effectively suppress the growth of cisplatin-resistant NPC43 cells (Fig. 6g). In summary, cisplatin and palbociclib could each potentially be used to treat patients who have developed resistance to the other drug.

## Conclusions

In conclusion, palbociclib effectively induced cell cycle arrest and growth suppression in NPC models derived from primary, recurrent and metastatic tumors. The addition of SAHA further potentiated the inhibitory effect of palbociclib. In contrast, the concurrent use of cisplatin and palbociclib is not recommended. NPC cells that developed tolerance to palbociclib remained sensitive to cisplatin, and vice versa. Together, our work provides relevant information for the planning of clinical trials and applications of palbociclib-involved regimens for the treatment of NPC.

## Supplementary Information

**Additional file 1:** **Figure S1.** IC_50_ values of palbociclib in different cancers as adapted from the Genomics of Drug Sensitivity in Cancer database [31] **A**. A mean Day-3 palbociclib IC_50_ of 35.7 μM was calculated from 770 cell lines corresponding to 32 types of cancer. The Day-3 IC_50_ of palbociclib was 49.2 μM in 26 head and neck cancer cell lines. **B**. Comparison of the Day-3 IC_50_ values of palbociclib for our NPC cell lines and those included in the databank. **Figure S2.** Quantification of protein expression in western blots of Fig. 1e. The grayscale value of each band in Fig. 1e was detected using MyImage software. The protein bands were normalized to the corresponding GAPDH level in each cell line and then compared with the normalized level in the control group. **Figure S3.** Cytostatic effect of palbociclib in three-dimensional cultures of NPC cell lines. Cells were allowed to form spheroids and treated with palbociclib at various doses. Three-dimensional spheroids of (**A**) C666–1 and (**B**) C17 NPC cells were grown in ultra-low attachment plates. The viability of spheroids was examined using the Cell Titer Glo assay. Dose–response curves were plotted for NPC spheroids after treatment with palbociclib for 3 and 5 days. Western blot analysis indicated the significant downregulation of RB (Ser780) phosphorylation and cyclin A expression in spheroids treated with palbociclib at concentrations greater than 0.2 μM. **Figure S4.** Expression of p16, RB, and cyclin D1 in NPC xenografts grown in mouse models. Although p16 was undetectable in all NPC models, RB, and cyclin D1 were detectable at various levels in all models. **Figure S5.** Comparison of the inhibitory effect of palbociclib on the growth of different xenografts in mice. The tumor growth curves (upper panel), final tumor volumes (lower left panel), and final tumor growth inhibition (lower right panel) of all xenografts in Fig. 2 are summarized to enable a comparison of drug efficacy. **Figure S6.** Enlarged IHC images of tissues as shown in Figure 2c. **Figure S7.** Analysis of Ki-67 expression cells in tumor sections. **A**. Whole-tumor sections were subjected to immunohistochemical analysis for Ki-67 and scanned using a Vectra Polaris imager. The images were imported into the Pheno-Chart 13 analysis program and divided into numerous 466 μm × 349 μm regions. **B**. Within each region, the software automatically compartmentalized each cell according to the nuclear stain. Blue spots represent the hematoxylin-stained nuclei, and brown spots represent the Ki-67–positive cells. **C.** After pooling all the data from each analyzed region, the percentage of Ki-67–positive cells was calculated as the number of positive DAB-stained cells / total number of tumor cells × 100%. **Figure S8.** Inhibition of NPC metastasis by palbociclib. Each NOD/SCID mouse was injected with 10^6^ C666–1 cells via the tail vein, and treatment was initiated 10 days later. **A**. All control mice developed NPC metastases in the lungs; in contrast, only one of four mice in the palbociclib treatment group developed lung metastases. In addition, the metastatic nodules in the lungs of the control group were larger than those in the lungs of the affected treatment group mouse. **B.** Solid tumor nodules in the control group lung tissues are indicated by blue arrows. **C**. Hematoxylin-eosin (H&E) staining of representative tumors from each treatment group. The tumor nodules among the air sacs in the lungs are indicated by blue arrows. **D**. Higher magnification of the H&E-stained slides reveals the densely packed tumor cells next to the air sacs in the lung tissues of control group mice. **Figure S9.** Enlarged IHC images of tissues as shown in Fig. 4b. **Figure S10.** Suppression of metabolic activity in Xeno23 tumors after combined treatment with palbociclib and SAHA. Micro-PET/MRI scans of Xeno23-bearing mice reveal the suppressed metabolic activity in tumors treated with palbociclib + SAHA. The mice received vehicle or combined treatment for 17 days. **Figure S11.** Co-treatment with palbociclib and SAHA did not promote cell differentiation or apoptosis relative to palbociclib or SAHA monotherapy. NPC43 cells were treated with 15-μM palbociclib or 5-μM SAHA alone or in combination for 6 or 24 h. The levels of proteins related to cell cycle, differentiation, and apoptosis in the treated cells were examined by western blotting. **Figure S12.** Lack of prominent activation of the EBV lytic cycle in NPC cells subjected to palbociclib or SAHA monotherapy or combination treatment. RNAscope was used to detect the expression of *BZLF1* (which encodes the early lytic protein, Zta) in C666–1 tumors isolated from mice in the control and treatment groups. Five random regions were selected from the slides corresponding to each treatment group. The numbers of cells that harbor positive signals for BZLF1 RNA were calculated using inForm analysis software. The numbers of positive BZLF1 hybridization signal were comparable between the tumors from all control and treatment groups. Fewer than 1% of all cells in the analyzed tumor areas were positive for *BZLF1*, indicating that EBV lytic reactivation was not a major factor in the cell death induced by the drug treatments. **Figure S13.** Assessment of autophagic flux and viability in NPC43 and C17 cells in response to palbociclib and SAHA monotherapy and combined treatment. **A.** Analysis of autophagic flux in NPC43 and C17 cells treated with palbociclib, SAHA, or palbociclib + SAHA and the autophagic flux inhibitor chloroquine (CQ, 25 μM) for 24 h. **B**. Bar chart of the fold changes in LC3-II in treated cells relative to the vehicle control as shown in Fig. A. **C.** Viability of NPC43 and C17 cells in response to the conditions described in **A**. **Figure S14.** Knockdown of the autophagy-related proteins BECN1 and ATG5 partially reversed the cytotoxic effects of palbociclib and SAHA in NPC cells. **A.** NPC43 cells were transfected with siRNAs specific for BECN1 or ATG5 and then treated with palbociclib and SAHA for 24 h. Cell viability was tested using a resazurin assay. **B**. Knockdown of ATG5 and beclin-1 protein expression after transfection with corresponding siRNAs was verified by western blotting. The normalized grayscale values corresponding to the protein band intensities are marked in red above the bands. **Figure S15.** Assessment of the autophagy levels in NPC43, C666–1 and C17 cell lines by visualizing the autophagic vacuoles with GFP-LC3II. The number of autophagic vacuole as indicated by the expression of GFP-LC3-II were counted and represented in the bar charts. Increased autophagic flux in cells treated by palbociclib (15 μM) and SAHA (5 μM) for 24 h was observed.

## Data Availability

All experimental data generated or analyzed during this study are included in this published article and its supplementary files.

## Abbreviations

NPC: Nasopharyngeal carcinoma
EBV: Epstein–Barr virus
NPE: Nasopharyngeal epithelial cells
SAHA: Suberanilohydroxamic acid
HDAC: Histone deacetylase
LAU: Laboratory Animal Unit
HKU: The Hong Kong University
NBF: Neutral buffered formalin
DMF: Dimethylformamide
SDS: Sodium dodecyl sulfate
PET: Positron emission tomography
MRI: Magnetic resonance imaging
SUV: Standardized uptake value
FDG: ^18^F-flurodeoxyglucose
KEGG: Kyoto Encyclopedia of Genes and Genomes
CPDB: ConsensusPathDB database
CQ: Chloroquine
PD-R: Palbociclib-resistant
AMPK5′: AMP-activated protein kinase
PP5: Protein phosphatase 5

## References

[1] Chen YP, Chan ATC, Le QT, Blanchard P, Sun Y, Ma J (2019). Nasopharyngeal carcinoma. Lancet (London, England).

[2] Lee VHF, Lam KO, Lee AWM, Lee AWM, Lung ML, Ng WT (2019). Chapter 10 - standard of Care for Nasopharyngeal Carcinoma (2018–2020). Nasopharyngeal carcinoma: academic press.

[3] Chan JYW, Lam TC, Ng WT, Lee AWM, Lung ML, Ng WT (2019). Chapter 13 - salvage of local recurrence. Nasopharyngeal carcinoma: academic press.

[4] Busson P, Ganem G, Flores P, Mugneret F, Clausse B, Caillou B (1988). Establishment and characterization of three transplantable EBV-containing nasopharyngeal carcinomas. Int J Cancer.

[5] Huang DP, Ho JH, Chan WK, Lau WH, Lui M (1989). Cytogenetics of undifferentiated nasopharyngeal carcinoma xenografts from southern Chinese. Int J Cancer.

[6] Cheung ST, Huang DP, Hui AB, Lo KW, Ko CW, Tsang YS (1999). Nasopharyngeal carcinoma cell line (C666-1) consistently harbouring Epstein-Barr virus. Int J Cancer.

[7] Chan SY, Choy KW, Tsao SW, Tao Q, Tang T, Chung GT (2008). Authentication of nasopharyngeal carcinoma tumor lines. Int J Cancer.

[8] Lin W, Yip YL, Jia L, Deng W, Zheng H, Dai W (2018). Establishment and characterization of new tumor xenografts and cancer cell lines from EBV-positive nasopharyngeal carcinoma. Nat Commun.

[9] Yip YL, Lin W, Deng W, Jia L, Lo KW, Busson P (2018). Establishment of a nasopharyngeal carcinoma cell line capable of undergoing lytic Epstein-Barr virus reactivation. Lab Investig.

[10] Li YY, Chung GT, Lui VW, To KF, Ma BB, Chow C (2017). Exome and genome sequencing of nasopharynx cancer identifies NF-kappaB pathway activating mutations. Nat Commun.

[11] Lin DC, Meng X, Hazawa M, Nagata Y, Varela AM, Xu L (2014). The genomic landscape of nasopharyngeal carcinoma. Nat Genet.

[12] Dai W, Zheng H, Cheung AK, Tang CS, Ko JM, Wong BW (2016). Whole-exome sequencing identifies MST1R as a genetic susceptibility gene in nasopharyngeal carcinoma. Proc Natl Acad Sci U S A.

[13] Tsang CM, Lui VWY, Bruce JP, Pugh TJ, Lo KW. Translational genomics of nasopharyngeal cancer. Semin Cancer Biol. 2019. 10.1016/j.semcancer.2019.09.006. 10.1016/j.semcancer.2019.09.006 31521748

[14] Zhang L, MacIsaac KD, Zhou T, Huang PY, Xin C, Dobson JR (2017). Genomic analysis of nasopharyngeal carcinoma reveals TME-based subtypes. Mol Cancer Res.

[15] Zheng H, Dai W, Cheung AK, Ko JM, Kan R, Wong BW (2016). Whole-exome sequencing identifies multiple loss-of-function mutations of NF-kappaB pathway regulators in nasopharyngeal carcinoma. Proc Natl Acad Sci U S A.

[16] Lo K-W, Huang DP, Lau K-M (1995). p16 gene alterations in nasopharyngeal carcinoma. Cancer Res.

[17] Lo KW, Cheung ST, Leung SF, van Hasselt A, Tsang YS, Mak KF (1996). Hypermethylation of the p16 gene in nasopharyngeal carcinoma. Cancer Res.

[18] Tsang R, Guan X, Hui A, Or Y, Takano H, To K (2005). Array-based comparative genomic hybridization analysis identified cyclin D1 as a target oncogene at 11q13.3 in nasopharyngeal carcinoma. Cancer Res.

[19] Tsang CM, Yip YL, Lo KW, Deng W, To KF, Hau PM (2012). Cyclin D1 overexpression supports stable EBV infection in nasopharyngeal epithelial cells. Proc Natl Acad Sci U S A.

[20] Finn RS, Dering J, Conklin D, Kalous O, Cohen DJ, Desai AJ (2009). PD 0332991, a selective cyclin D kinase 4/6 inhibitor, preferentially inhibits proliferation of luminal estrogen receptor-positive human breast cancer cell lines in vitro. Breast Cancer Res.

[21] Finn RS, Martin M, Rugo HS, Jones S, Im S-A, Gelmon K (2016). Palbociclib and letrozole in advanced breast cancer. New Engl J Med.

[22] Bollard J, Miguela V, Ruiz de Galarreta M, Venkatesh A, Bian CB, Roberto MP (2016). Palbociclib (P-7766) - Palbociclib (PD-0332991), a selective CDK4/6 inhibitor, restricts tumour growth in preclinical models of hepatocellular carcinoma. Gut.

[23] Konecny GE, Winterhoff B, Kolarova T, Qi J, Manivong K, Dering J (2011). Expression of p16 and retinoblastoma determines response to CDK4/6 inhibition in ovarian cancer. Clin Cancer Res.

[24] Katsumi Y, Iehara T, Miyachi M, Yagyu S, Tsubai-Shimizu S, Kikuchi K (2011). Sensitivity of malignant rhabdoid tumor cell lines to PD 0332991 is inversely correlated with p16 expression. Biochem Biophys Res Commun.

[25] Cen L, Carlson BL, Schroeder MA, Ostrem JL, Kitange GJ, Mladek AC (2012). p16-Cdk4-Rb axis controls sensitivity to a cyclin-dependent kinase inhibitor PD0332991 in glioblastoma xenograft cells. Neuro-Oncology.

[26] Xu WS, Parmigiani RB, Marks PA (2007). Histone deacetylase inhibitors: molecular mechanisms of action. Oncogene.

[27] Li HM, Man C, Jin Y, Deng W, Yip YL, Feng HC (2006). Molecular and cytogenetic changes involved in the immortalization of nasopharyngeal epithelial cells by telomerase. In J Cancer.

[28] Tsao SW, Wang X, Liu Y, Cheung YC, Feng H, Zheng Z (2002). Establishment of two immortalized nasopharyngeal epithelial cell lines using SV40 large T and HPV16E6/E7 viral oncogenes. Biochim Biophys Acta.

[29] Zhang J, Jia L, Liu T, Yip YL, Tang WC, Lin W (2019). mTORC2-mediated PDHE1alpha nuclear translocation links EBV-LMP1 reprogrammed glucose metabolism to cancer metastasis in nasopharyngeal carcinoma. Oncogene.

[30] Kim D, Langmead B, Salzberg SL (2015). HISAT: a fast spliced aligner with low memory requirements. Nat Methods.

[31] Pertea M, Kim D, Pertea GM, Leek JT, Salzberg SL (2016). Transcript-level expression analysis of RNA-seq experiments with HISAT. StringTie and Ballgown Nat Protoc.

[32] Robinson MD, McCarthy DJ, Smyth GK (2010). edgeR: a bioconductor package for differential expression analysis of digital gene expression data. Bioinformatics.

[33] Iorio F, Knijnenburg TA, Vis DJ, Bignell GR, Menden MP, Schubert M (2016). A landscape of pharmacogenomic interactions in cancer. Cell.

[34] Hui KF, Ho DN, Tsang CM, Middeldorp JM, Tsao GS, Chiang AK (2012). Activation of lytic cycle of Epstein-Barr virus by suberoylanilide hydroxamic acid leads to apoptosis and tumor growth suppression of nasopharyngeal carcinoma. Int J Cancer.

[35] Chou TC, Talalay P (1984). Quantitative analysis of dose-effect relationships: the combined effects of multiple drugs or enzyme inhibitors. Adv Enzym Regul.

[36] Chou T-C (2010). Drug combination studies and their synergy quantification using the Chou-Talalay method. Cancer Res.

[37] Mizushima N, Yoshimori T (2007). How to interpret LC3 immunoblotting. Autophagy.

[38] Ngan RKC, Li KWS, Chow JCH, Yip TTC, Lee AWM, Lung ML, Ng WT (2019). Chapter 14 - Management of Metastatic Nasopharyngeal Carcinoma. Nasopharyngeal carcinoma: academic press.

[39] Xu C, Chen YP, Ma J (2016). Clinical trials in nasopharyngeal carcinoma-past, present and future. Chin Clin Oncol.

[40] NIH. 2020 Clinical Trials.gov. <https://clinicaltrials.gov/ct2/results?cond=Nasopharyngeal+Carcinoma&term=&type=Intr&rslt=&recrs=b&recrs=a&recrs=f&recrs=d&recrs=c&age_v=&gndr=&intr=&titles=&outc=&spons=&lead=&id=&cntry=&state=&city=&dist=&locn=&phase=2&rsub=&strd_s=&strd_e=&prcd_s=&prcd_e=&sfpd_s=&sfpd_e=&rfpd_s=&rfpd_e=&lupd_s=&lupd_e=&sort=>.

[41] Han B, Li K, Wang Q, Zhang L, Shi J, Wang Z (2018). Effect of anlotinib as a third-line or further treatment on overall survival of patients with advanced non–small cell lung cancer: the ALTER 0303 phase 3 randomized clinical trial. JAMA Oncol.

[42] Fang W, Yang Y, Ma Y, Hong S, Lin L, He X (2018). Camrelizumab (SHR-1210) alone or in combination with gemcitabine plus cisplatin for nasopharyngeal carcinoma: results from two single-arm, phase 1 trials. Lancet Oncol.

[43] Qin S, Finn RS, Kudo M, Meyer T, Vogel A, Ducreux M (2019). RATIONALE 301 study: tislelizumab versus sorafenib as first-line treatment for unresectable hepatocellular carcinoma. Future Oncol.

[44] Ramakrishnan MS, Eswaraiah A, Crombet T, Piedra P, Saurez G, Iyer H (2009). Nimotuzumab, a promising therapeutic monoclonal for treatment of tumors of epithelial origin. mAbs.

[45] Yip YL, Lin WT, Deng W, Tsang CM, Tsao SW, Lee AWM, Lung ML, Ng WT (2019). Chapter 5 - establishment of nasopharyngeal carcinoma cell lines, patient-derived Xenografts, and immortalized nasopharyngeal epithelial cell lines for nasopharyngeal carcinoma and Epstein–Barr virus infection studies. Nasopharyngeal carcinoma: academic press.

[46] Yang C, Boyson CA, Di Liberto M, Huang X, Hannah J, Dorn DC (2015). CDK4/6 inhibitor PD 0332991 sensitizes acute myeloid leukemia to cytarabine-mediated cytotoxicity. Cancer Res.

[47] Menu E, Garcia J, Huang X, Di Liberto M, Toogood PL, Chen I (2008). A novel therapeutic combination using PD 0332991 and bortezomib: study in the 5T33MM myeloma model. Cancer Res.

[48] Teh JL, Purwin TJ, Greenawalt EJ, Chervoneva I, Goldberg A, Davies MA (2016). An in vivo reporter to quantitatively and temporally analyze the effects of CDK4/6 inhibitor-based therapies in melanoma. Cancer Res.

[49] Qin G, Xu F, Qin T, Zheng Q, Shi D, Xia W (2015). Palbociclib inhibits epithelial-mesenchymal transition and metastasis in breast cancer via c-Jun/COX-2 signaling pathway. Oncotarget.

[50] Wong CH, Ma BBY, Hui CWC, Lo KW, Hui EP, Chan ATC (2018). Preclinical evaluation of ribociclib and its synergistic effect in combination with alpelisib in non-keratinizing nasopharyngeal carcinoma. Sci Rep.

[51] Hsieh WS, Soo R, Peh BK, Loh T, Dong D, Soh D (2009). Pharmacodynamic effects of seliciclib, an orally administered cell cycle modulator, in undifferentiated nasopharyngeal cancer. Clin Cancer Res.

[52] Jiao X-D, Liu K, Qin B-D, Wu Y, Lin M-Q, Liu J (2019). Palbociclib for the treatment of metastatic nasopharyngeal carcinoma with CDK4 amplification: a case report. JCO Precis Oncol.

[53] Franco J, Witkiewicz AK, Knudsen ES (2014). CDK4/6 inhibitors have potent activity in combination with pathway selective therapeutic agents in models of pancreatic cancer. Oncotarget.

[54] Fassl A, Sicinski P (2020). Chemotherapy and CDK4/6 inhibition in cancer treatment: timing is everything. Cancer Cell.

[55] Florea AM, Busselberg D (2011). Cisplatin as an anti-tumor drug: cellular mechanisms of activity, drug resistance and induced side effects. Cancers (Basel).

[56] Keshelava N, Houghton PJ, Morton CL, Lock RB, Carol H, Keir ST (2009). Initial testing (stage 1) of vorinostat (SAHA) by the pediatric preclinical testing program. Pediatr Blood Cancer.

[57] Marks PA (2007). Discovery and development of SAHA as an anticancer agent. Oncogene.

[58] Ververis K, Hiong A, Karagiannis TC, Licciardi PV (2013). Histone deacetylase inhibitors (HDACIs): multitargeted anticancer agents. Biologics.

[59] Lee JH, Choy ML, Ngo L, Foster SS, Marks PA (2010). Histone deacetylase inhibitor induces DNA damage, which normal but not transformed cells can repair. Proc Natl Acad Sci U S A.

[60] Hui KF, Lam BH, Ho DN, Tsao SW, Chiang AK (2013). Bortezomib and SAHA synergistically induce ROS-driven caspase-dependent apoptosis of nasopharyngeal carcinoma and block replication of Epstein-Barr virus. Mol Cancer Ther.

[61] Lin L, Baehrecke EH (2015). Autophagy, cell death, and cancer. Mol Cell Oncol.

[62] Nam HY, Han MW, Chang HW, Lee YS, Lee M, Lee HJ (2013). Radioresistant cancer cells can be conditioned to enter senescence by mTOR inhibition. Cancer Res.

[63] Hsieh FS, Chen YL, Hung MH, Chu PY, Tsai MH, Chen LJ (2017). Palbociclib induces activation of AMPK and inhibits hepatocellular carcinoma in a CDK4/6-independent manner. Mol Oncol.

[64] Shao Y, Gao Z, Marks PA, Jiang X (2004). Apoptotic and autophagic cell death induced by histone deacetylase inhibitors. Proc Natl Acad Sci U S A.

[65] Yamamoto S, Tanaka K, Sakimura R, Okada T, Nakamura T, Li Y (2008). Suberoylanilide hydroxamic acid (SAHA) induces apoptosis or autophagy-associated cell death in chondrosarcoma cell lines. Anticancer Res.

[66] Yang C, Li Z, Bhatt T, Dickler M, Giri D, Scaltriti M (2017). Acquired CDK6 amplification promotes breast cancer resistance to CDK4/6 inhibitors and loss of ER signaling and dependence. Oncogene.

[67] Pandey K, An HJ, Kim SK, Lee SA, Kim S, Lim SM (2019). Molecular mechanisms of resistance to CDK4/6 inhibitors in breast cancer: a review. Int J Cancer.

