# Supplementary material for: Therapeutic evaluation of palbociclib and its compatibility with other chemotherapies for primary and recurrent nasopharyngeal carcinoma
Source: J Exp Clin Cancer Res. 2020 Nov 26;39:262. doi: 10.1186/s13046-020-01763-z (PMC7690146; doi:10.1186/s13046-020-01763-z)

Supplementary Figure S1

A

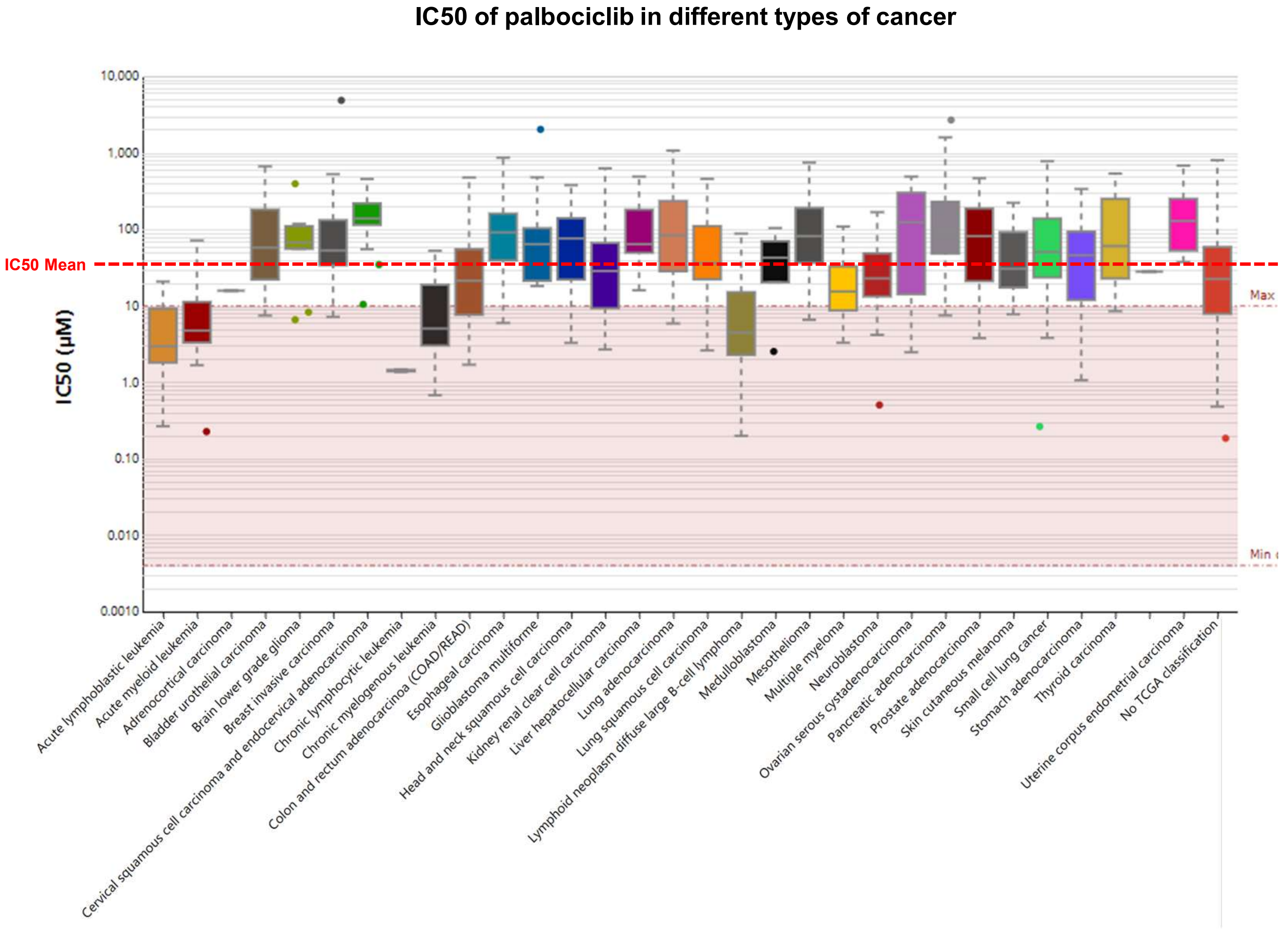

	PAN cancer	Head and Neck Cancer	Nasopharyngeal Carcinoma		
Cell Line	770 cell line	26 cell line	C666-1	C17	NPC43
IC50 (μM)（Day3 ）	35.7	49.2	22.36	20.23	24.63

Supplementary Figure S2

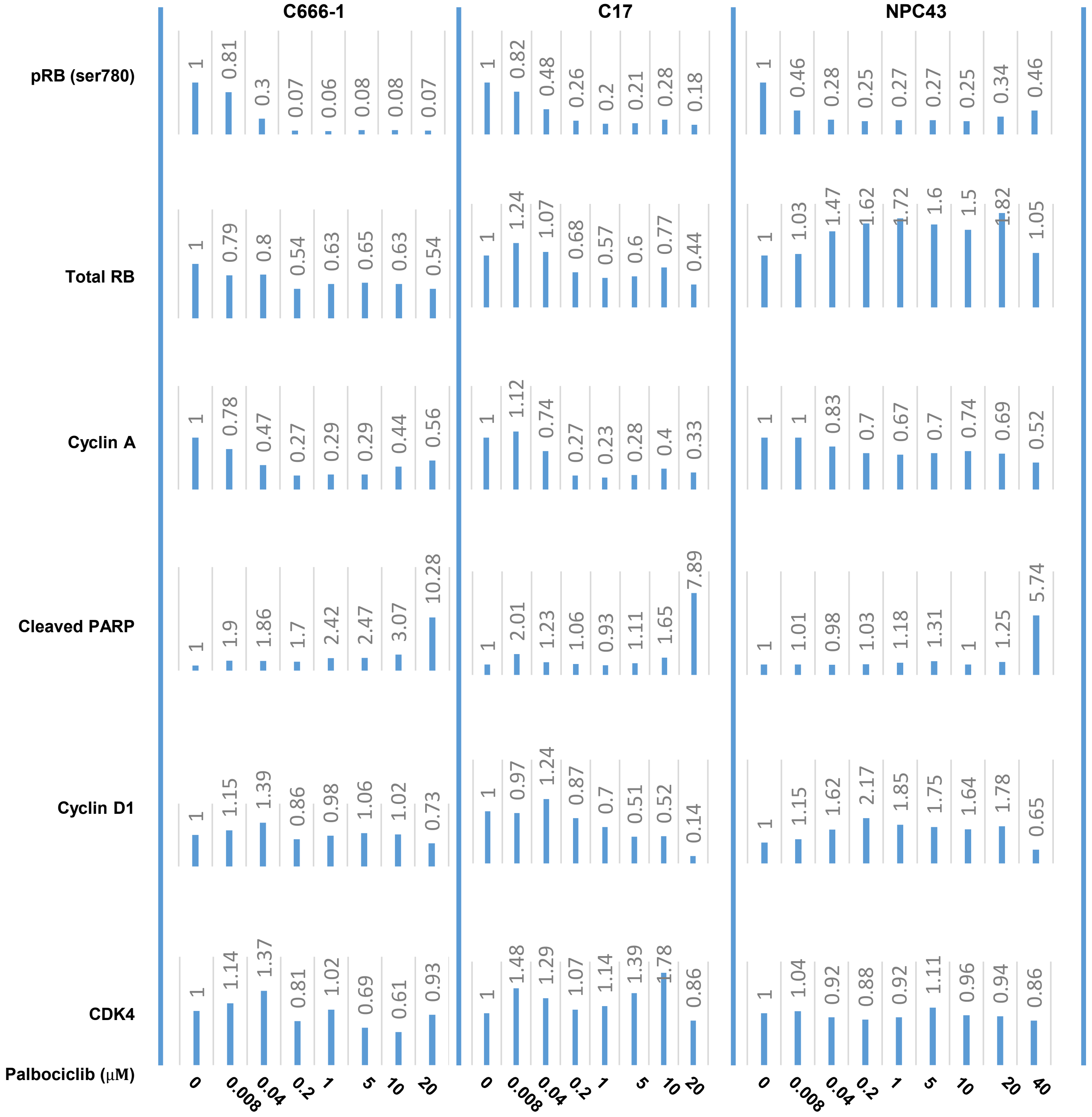

Supplementary Figure S3

A

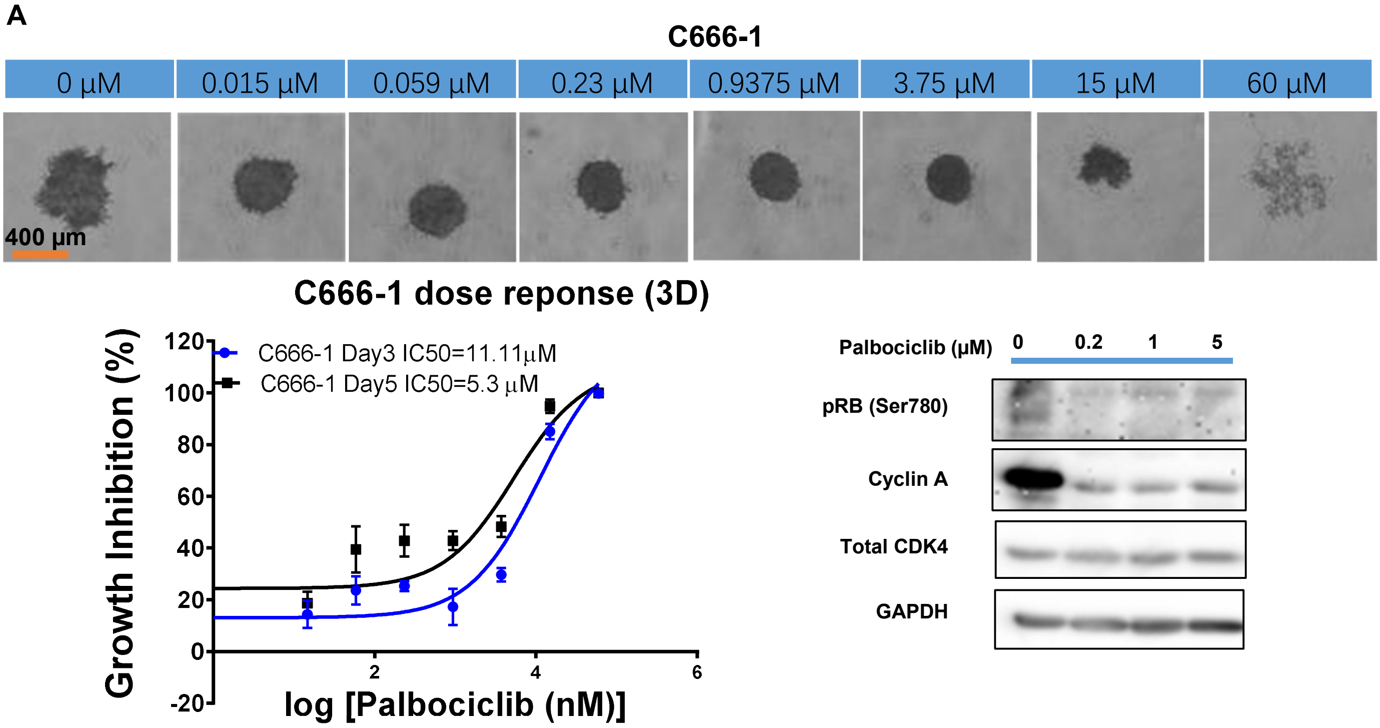

B

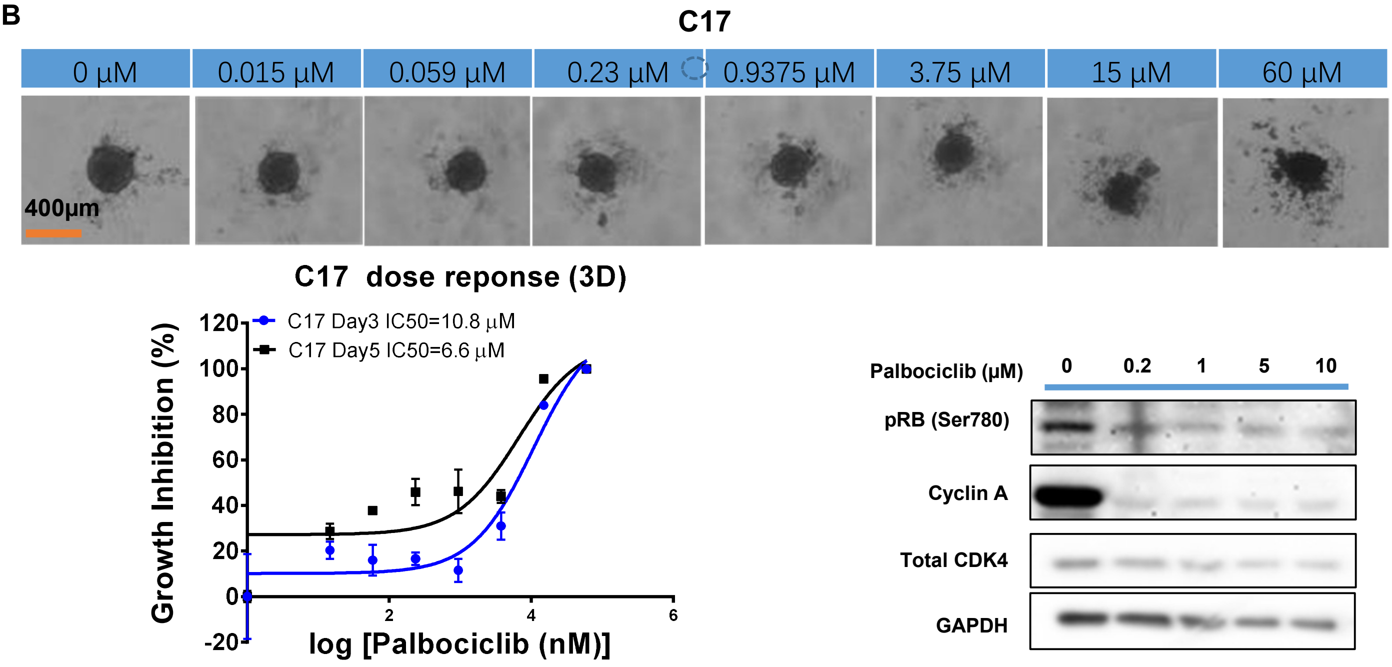

Supplementary Figure S4

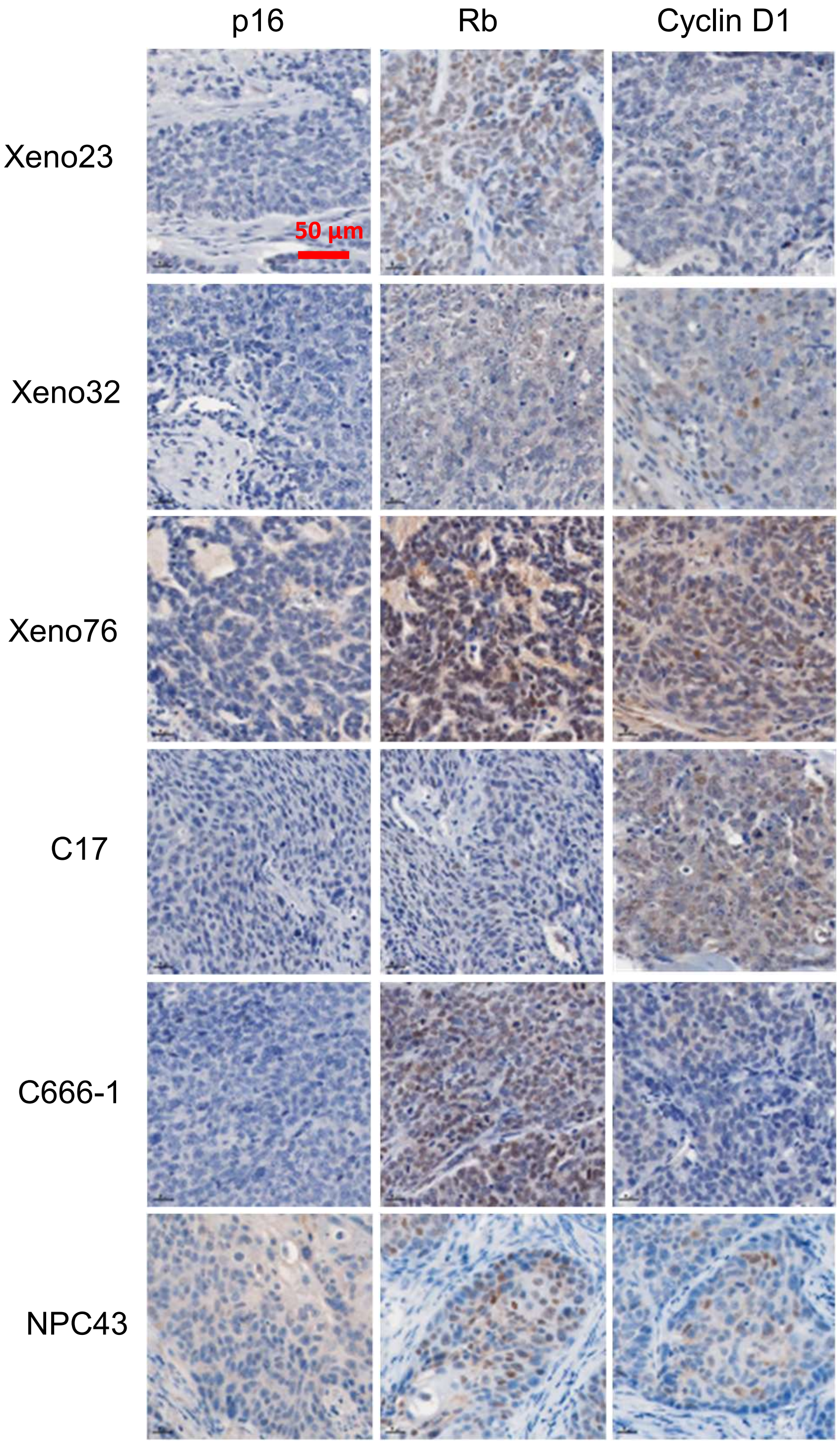

	p16	Rb	Cyclin D1
Xeno23	−	++	+
Xeno32	−	+	+
Xeno76	−	+++	+++
C17	−	+	+++
C666-1	−	+++	+
NPC43	−	++	++

Supplementary Figure S5

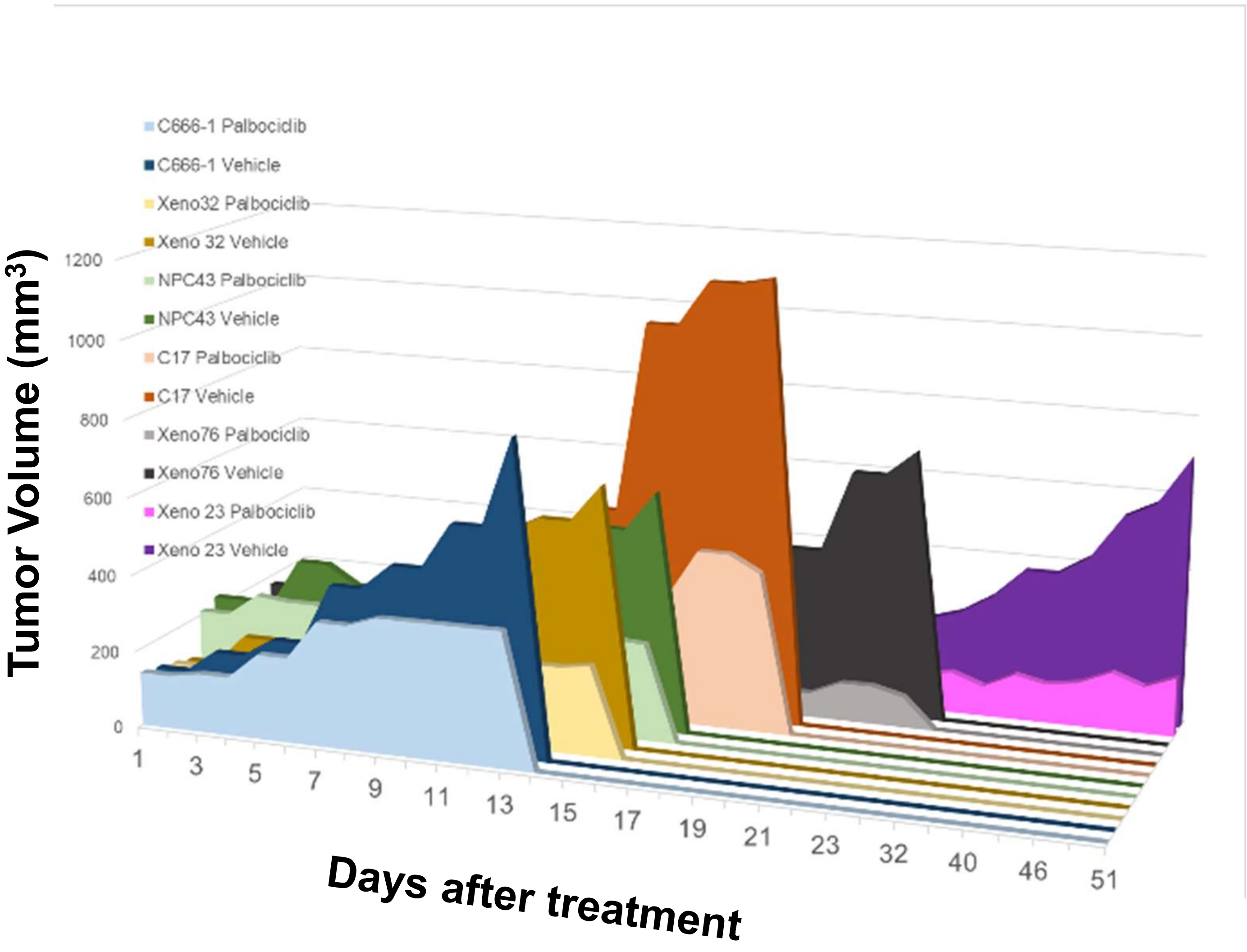

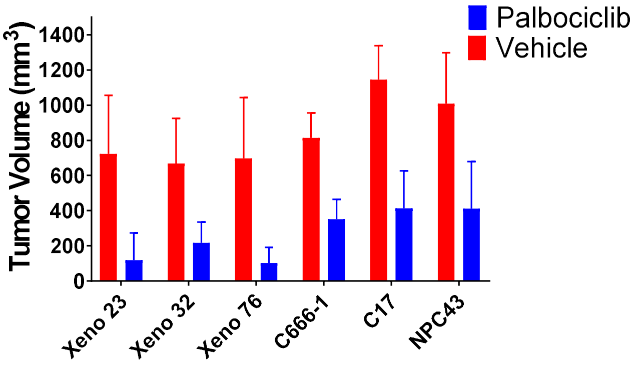

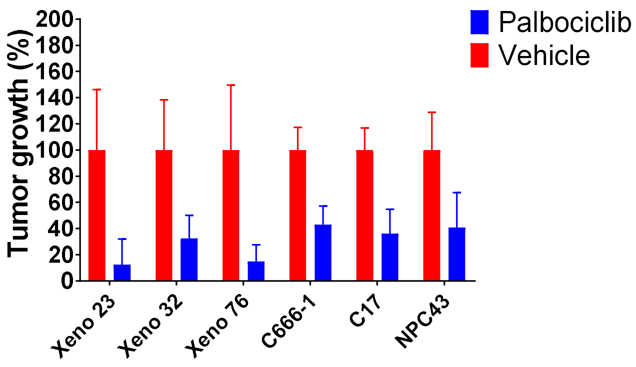

Supplementary Figure S6

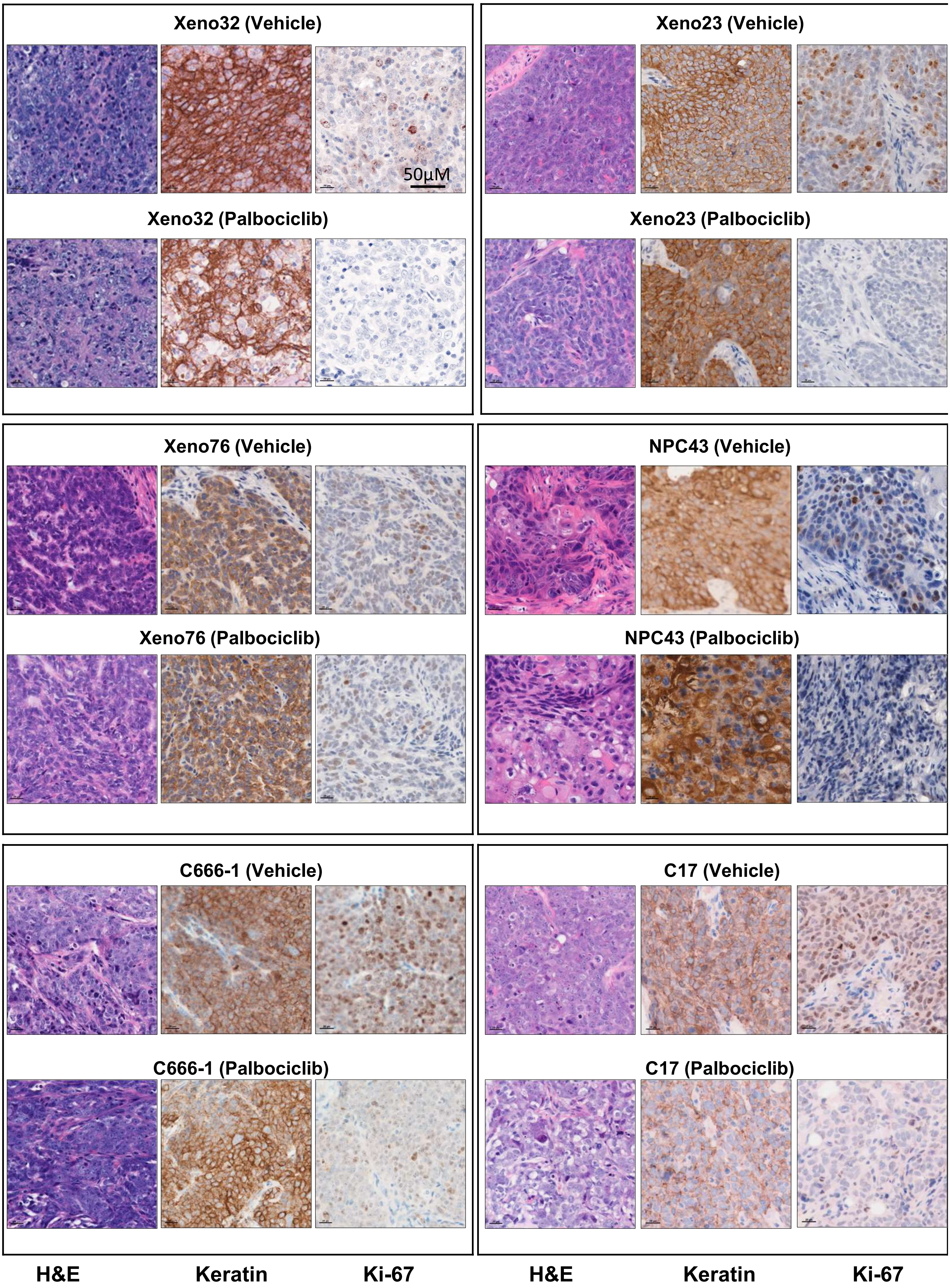

Supplementary Figure S7

A

B

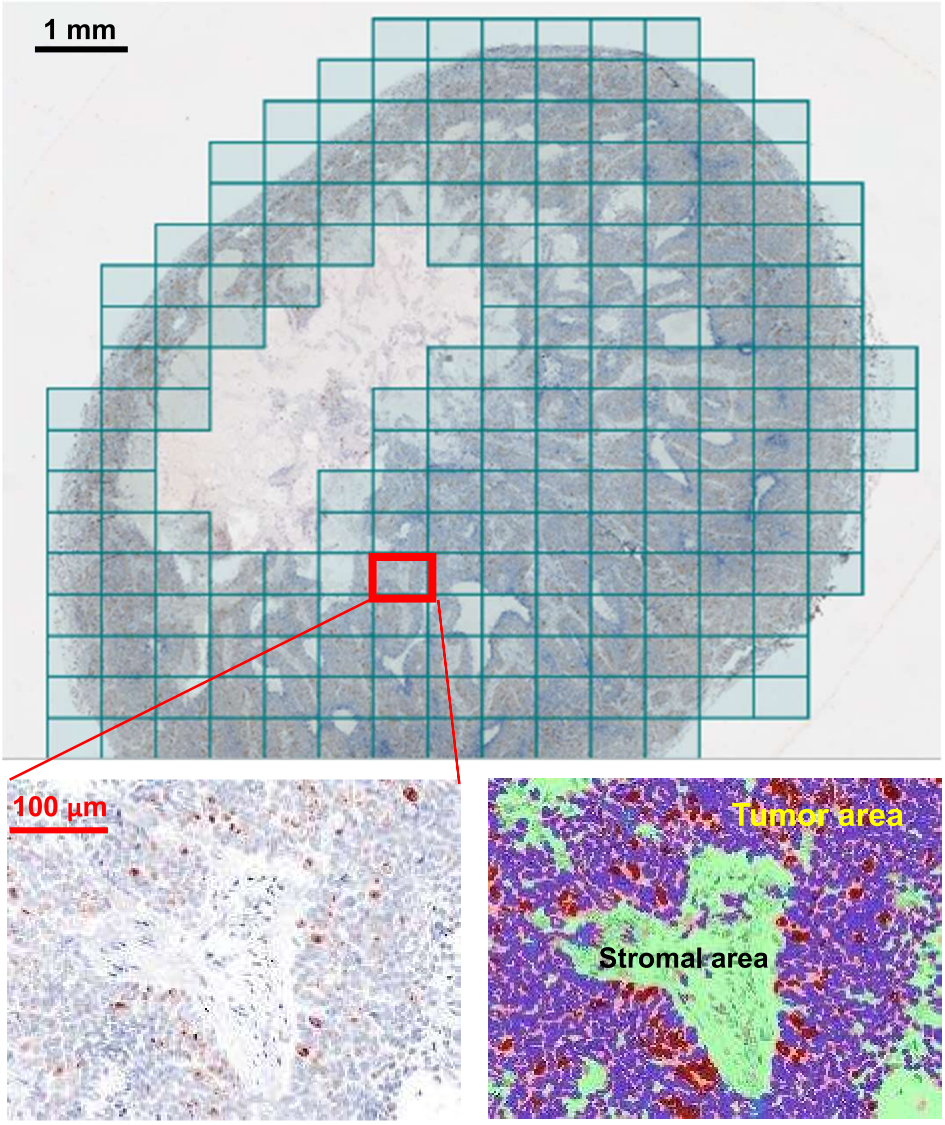

C

Number of tumor cells	Ki-67 positive cell (%)
1247	15.78%

Supplementary Figure S8

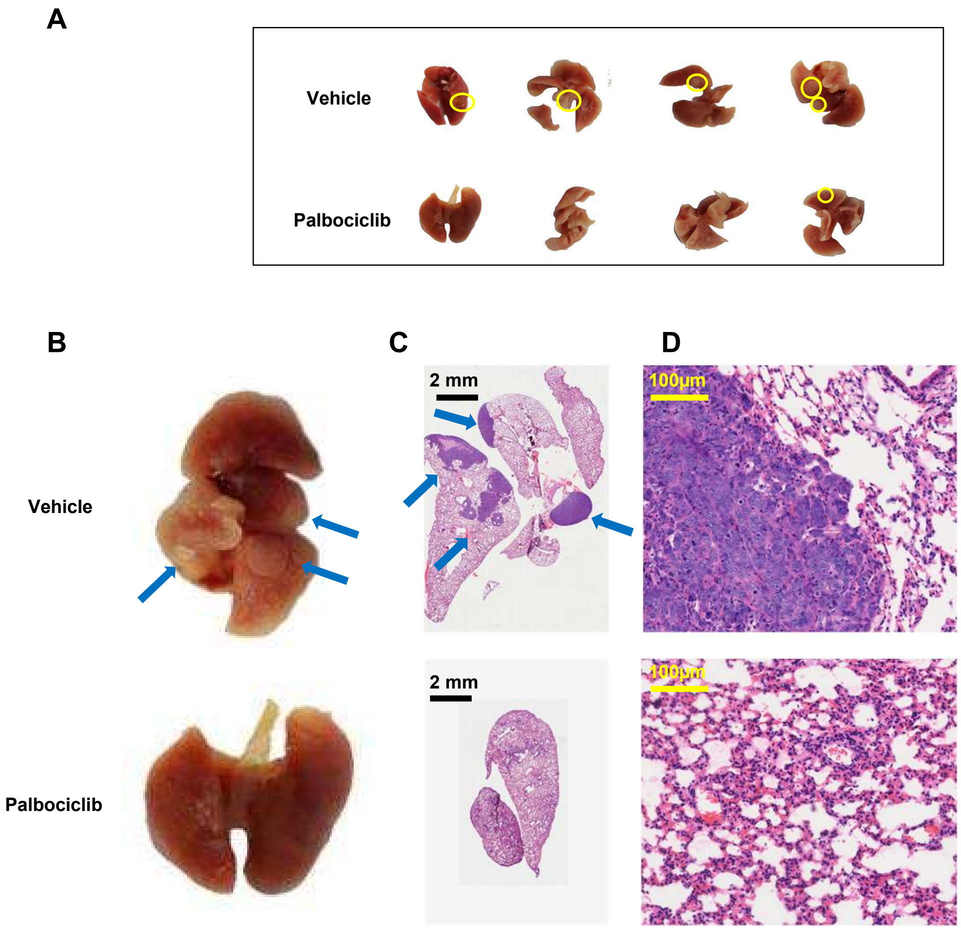

Supplementary Figure S9

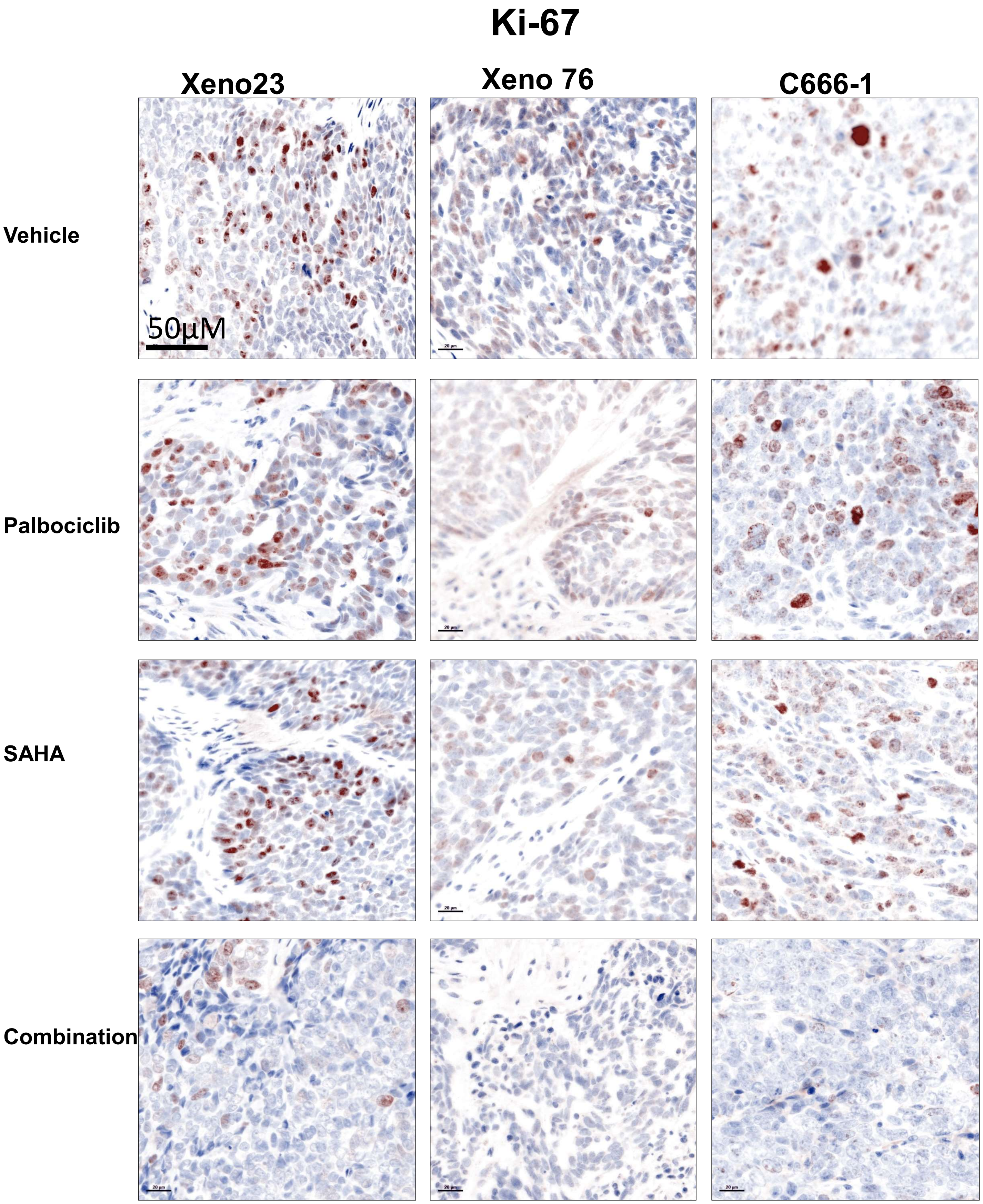

Supplementary Figure S10

Xeno23

Vehicle **Combination**

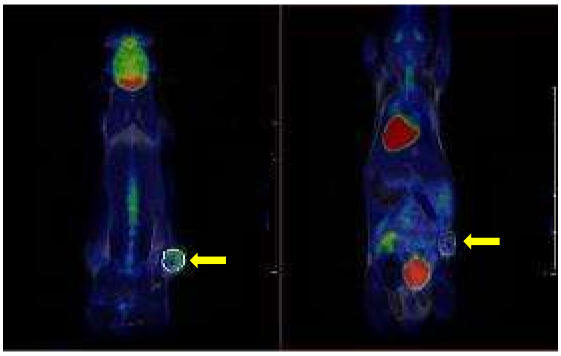

Xeno23	SUV mean<br>(Normalized by liver metabolism)
Vehicle	0.76
PD+SAHA	0.09

Supplementary Figure S11

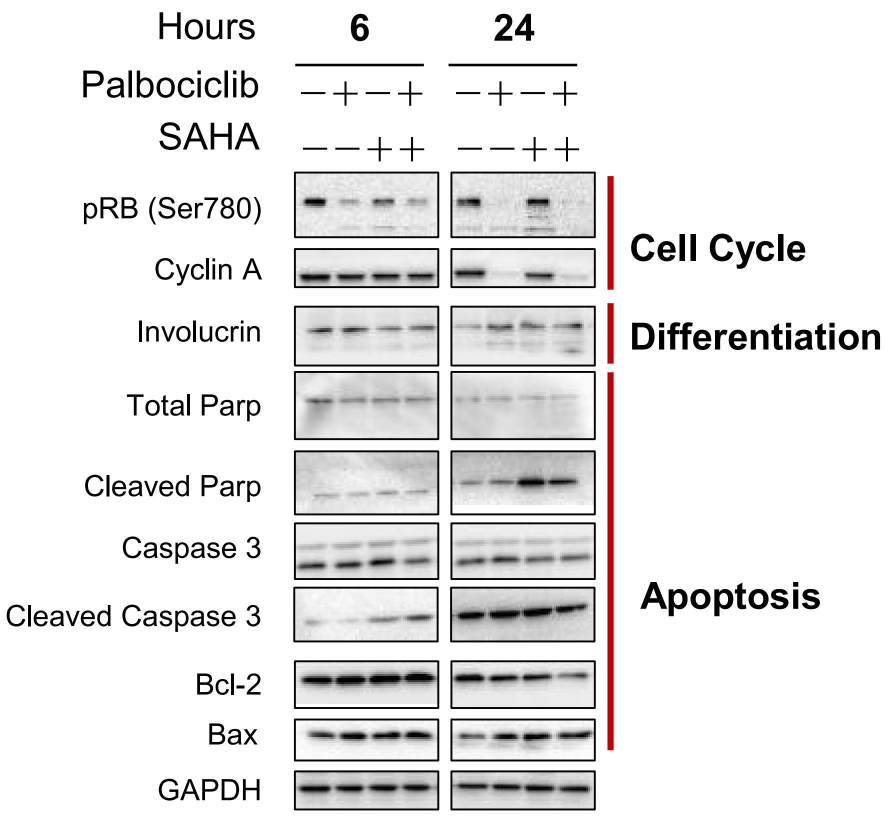

Supplementary Figure S12

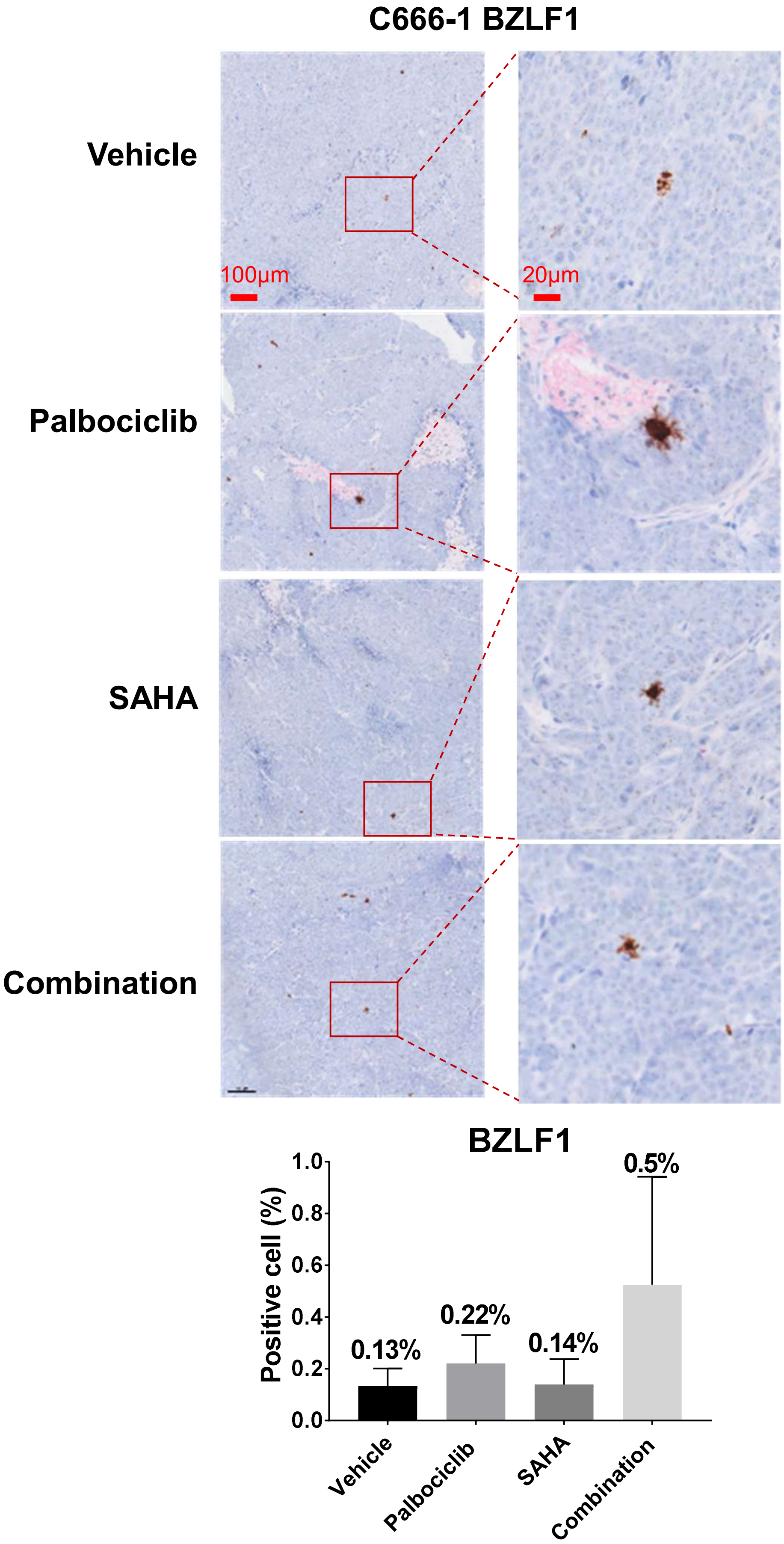

Supplementary Figure S13

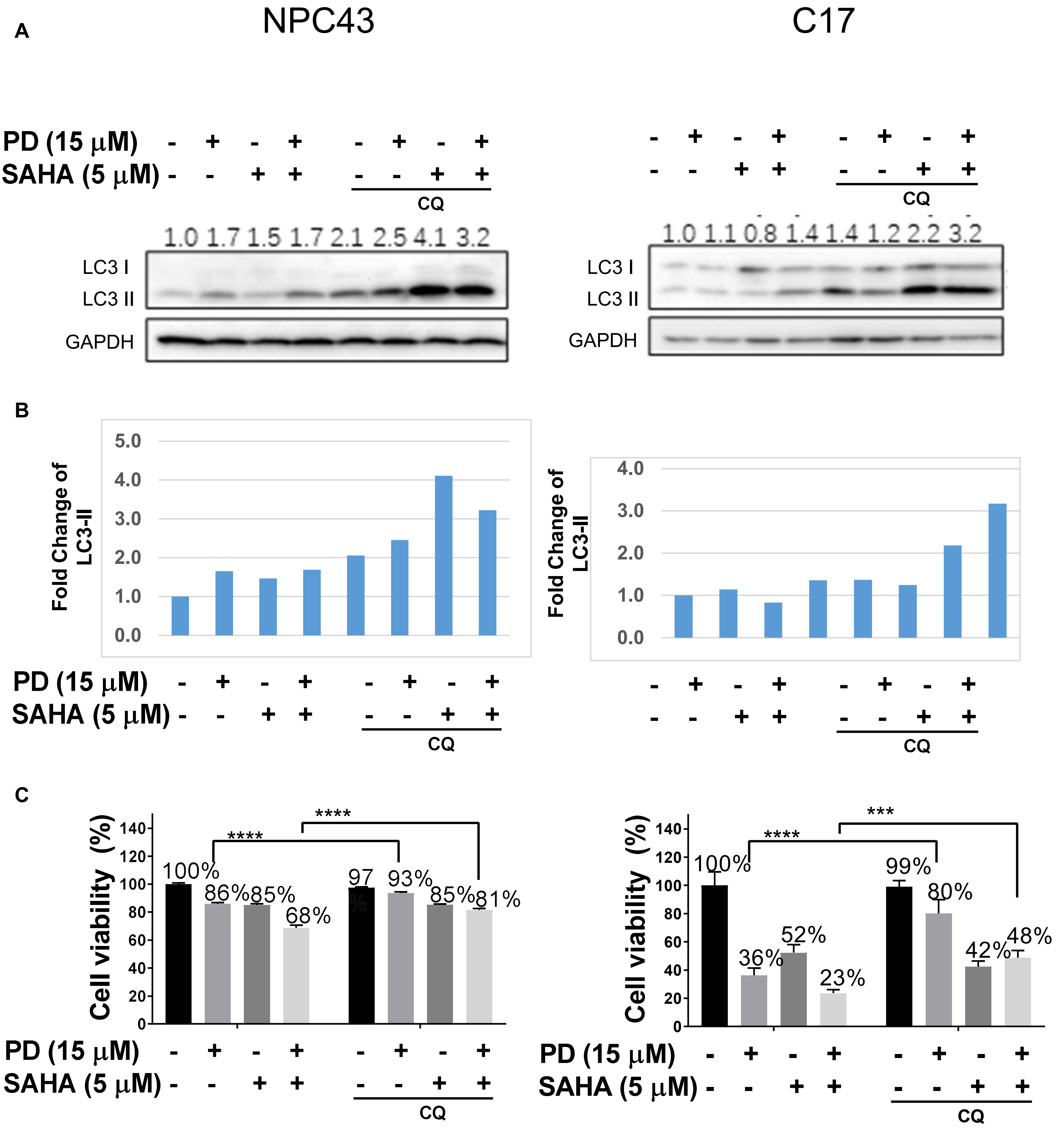

Supplementary Figure S14

A

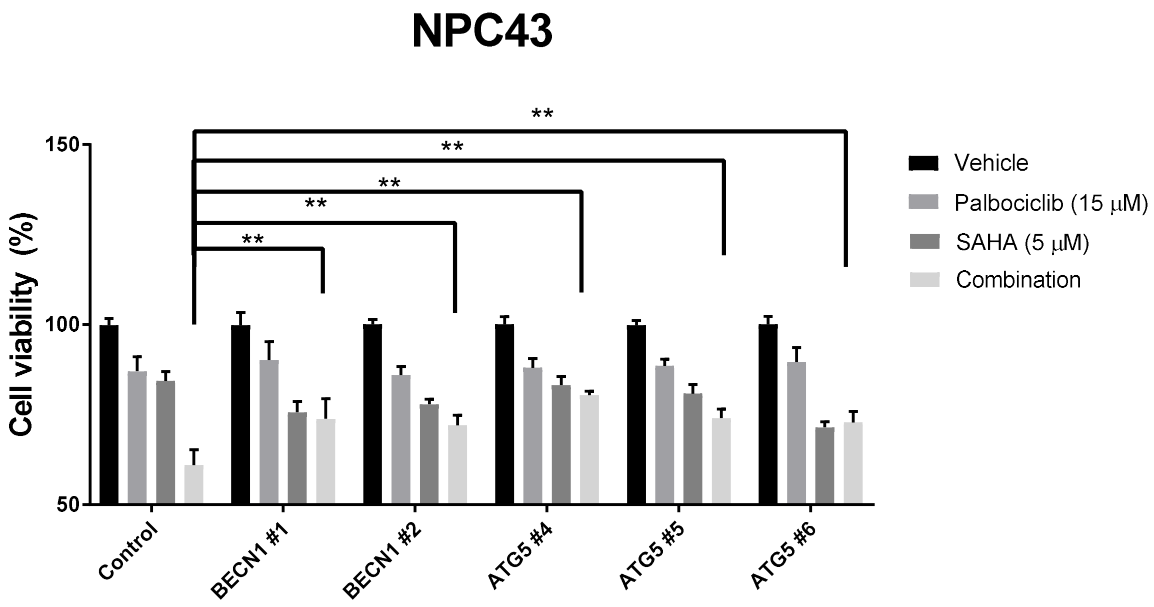

B

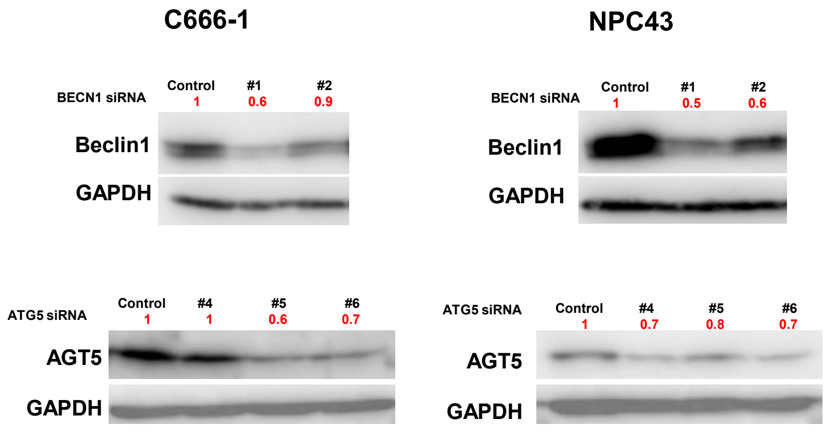

Supplementary Figure S15

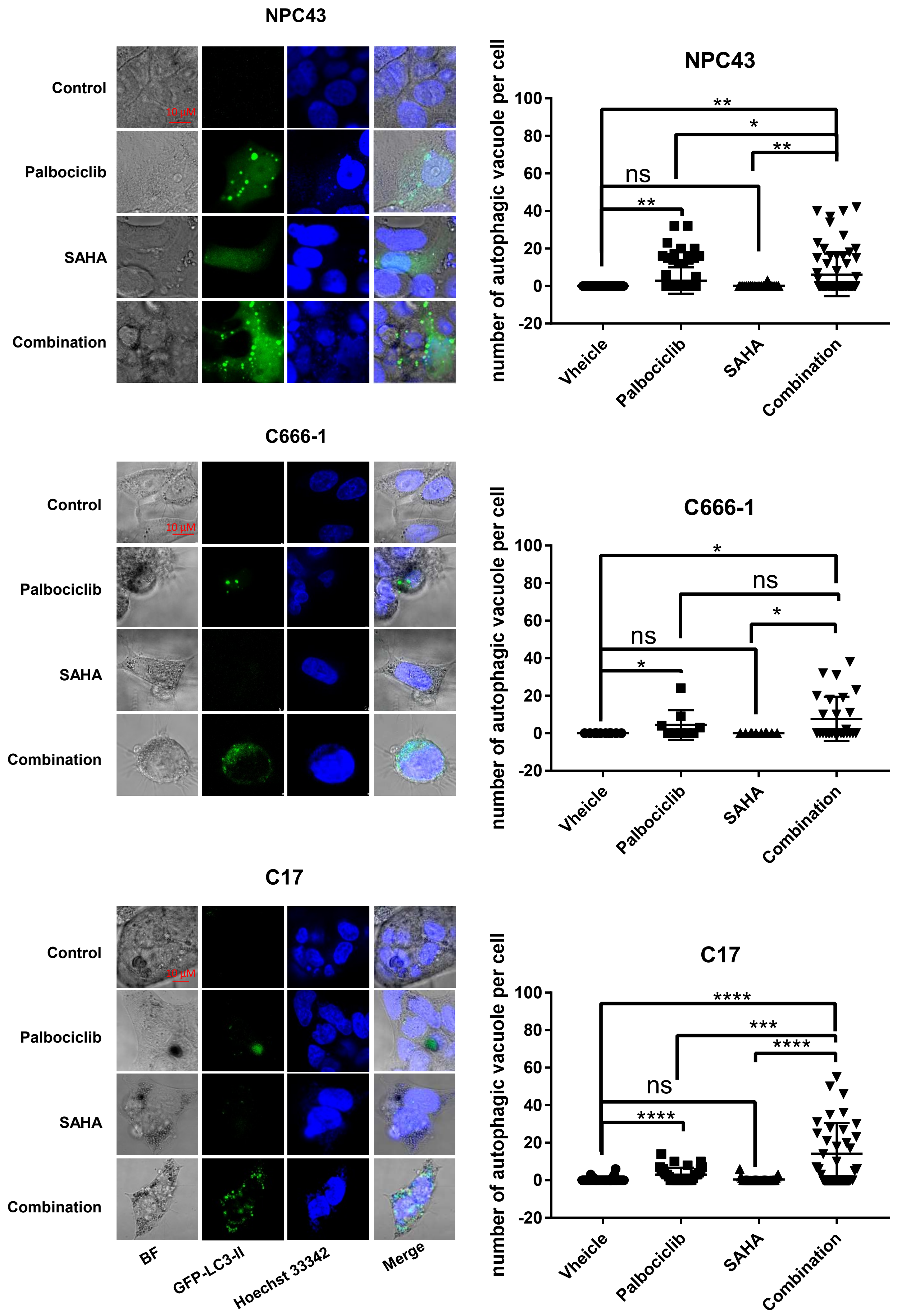

Supplement: Supplementary file 1 — Additional file 1: Figure S1. IC50 values of palbociclib in different cancers as adapted from the Genomics of Drug Sensitivity in Cancer database [31] A. A mean Day-3 palbociclib IC50 of 35.7 μM was calculated from 770 cell lines corresponding to 32 types of cancer. The Day-3 IC50 of palbociclib was 49.2 μM in 26 head and neck cancer cell lines. B. Comparison of the Day-3 IC50 values of palbociclib for our NPC cell lines and those included in the databank. Figure S2. Quantification of protein expression in western blots of Fig. 1e. The grayscale value of each band in Fig. 1e was detected using MyImage software. The protein bands were normalized to the corresponding GAPDH level in each cell line and then compared with the normalized level in the control group. Figure S3. Cytostatic effect of palbociclib in three-dimensional cultures of NPC cell lines. Cells were allowed to form spheroids and treated with palbociclib at various doses. Three-dimensional spheroids of (A) C666–1 and (B) C17 NPC cells were grown in ultra-low attachment plates. The viability of spheroids was examined using the Cell Titer Glo assay. Dose–response curves were plotted for NPC spheroids after treatment with palbociclib for 3 and 5 days. Western blot analysis indicated the significant downregulation of RB (Ser780) phosphorylation and cyclin A expression in spheroids treated with palbociclib at concentrations greater than 0.2 μM. Figure S4. Expression of p16, RB, and cyclin D1 in NPC xenografts grown in mouse models. Although p16 was undetectable in all NPC models, RB, and cyclin D1 were detectable at various levels in all models. Figure S5. Comparison of the inhibitory effect of palbociclib on the growth of different xenografts in mice. The tumor growth curves (upper panel), final tumor volumes (lower left panel), and final tumor growth inhibition (lower right panel) of all xenografts in Fig. 2 are summarized to enable a comparison of drug efficacy. Figure S6. Enlarged IHC images of tissues as [file 13046_2020_1763_MOESM1_ESM.pdf]
